# *Lactobacillus johnsonii*-derived extracellular vesicles restore mucosal immunity via taurine-linked Th17/Treg and IgA/IgG regulation in colitis

**DOI:** 10.1186/s12951-025-03702-6

**Published:** 2025-09-29

**Authors:** Hailan Zhao, Ningning Yue, Zhiliang Mai, Yuan Zhang, Chengmei Tian, Chen Kong, Longbin Huang, Ruiyue Shi, Yujie Liang, Jun Yao, Yuqiang Nie, Defeng Li, Biao Nie, Lisheng Wang

**Affiliations:** 1 Department of Gastroenterology, Shenzhen People’s Hospital (The First Affiliated Hospital, Southern University of Science and Technology; The Second Clinical Medical College, Jinan University), Shenzhen, 518020 Guangdong China; 2 The First Affiliated Hospital, Jinan University, Guangzhou, 510630 Guangdong China; 3 Department of Gastroenterology and Hepatology, Guangzhou First People’s Hospital, The Second Affiliated Hospital, School of Medicine, South China University of Technology, Guangzhou, 510006 Guangdong China; 4 Department of Medical Administration, Huizhou Institute of Occupational Diseases Control and Prevention, Huizhou, 516008 Guangdong China; 5 Department of Emergency, Shenzhen People’s Hospital (The First Affiliated Hospital, Southern University of Science and Technology; The Second Clinical Medical College, Jinan University), Shenzhen, 518020 Guangdong China; 6 Department of Child and Adolescent Psychiatry, Shenzhen Institute of Mental Health, Shenzhen Kangning Hospital, Shenzhen, 518020 Guangdong China

**Keywords:** *Lactobacillus johnsonii*, Extracellular vesicles, Mucosal immune responses, Immunoglobulin-coated bacteria, Amino acid metabolism, Gut barrier, Ulcerative colitis

## Abstract

**Background:**

Aberrant mucosal immune responses to gut microbiota contribute to inflammatory bowel disease (IBD), yet the mechanisms linking immunoglobulin-coated bacteria to mucosal inflammation remain unclear. Microbial extracellular vesicles (EVs) have emerged as potential modulators of host–microbiota interactions, metabolism, and immunity. This study examined how *Lactobacillus johnsonii*-derived EVs influence gut microbiota, amino acid metabolism, mucosal T cell polarization—particularly the Th17/Treg balance—and immunoglobulin transport in experimental colitis.

**Results:**

Flow cytometry of fecal samples from IBD patients revealed increased IgA-, IgG-, and IgM-coated bacteria. Colonic expression of PIGR and FcRn was also elevated. *L. johnsonii* was depleted in ulcerative colitis, whereas Proteobacteria and *Escherichia*_*Shigella* were enriched. EVs displayed high structural integrity, gastrointestinal stability, and enhanced accumulation in inflamed colon. In DSS-induced colitis mice, both *L. johnsonii* and EVs alleviated inflammation, improved histology, reduced pro-inflammatory cytokines, decreased Th17 cells, increased Treg cells, and restored the Th17/Treg balance. These interventions also reduced IgA-, IgG-, and IgM-coated bacteria, lowered fecal immunoglobulins without affecting systemic levels, and downregulated PIGR and FcRn. Multi-omics analyses showed that EVs reshaped gut microbiota, enriched taurine-associated taxa (Lactobacillales, Lactobacillaceae, *Lactobacillus murinus*), and elevated the immunoregulatory metabolite taurine, which was linked to sulfur metabolism and epithelial homeostasis. Taurine supplementation reproduced EV effects, including reduced inflammation, improved barrier integrity, Th17/Treg rebalancing, and suppression of PIGR and FcRn.

**Conclusions:**

*L. johnsonii*-derived EVs restore mucosal immune balance in colitis through a coordinated EV–taurine–Th17/Treg–PIGR/FcRn–IgA/IgG axis. By integrating microbiota remodeling, metabolic regulation, and immune modulation—and outperforming the parent bacterium in stability, colonic enrichment, and breadth of effect—these EVs represent promising next-generation biologics for IBD therapy.

**Graphical abstract:**

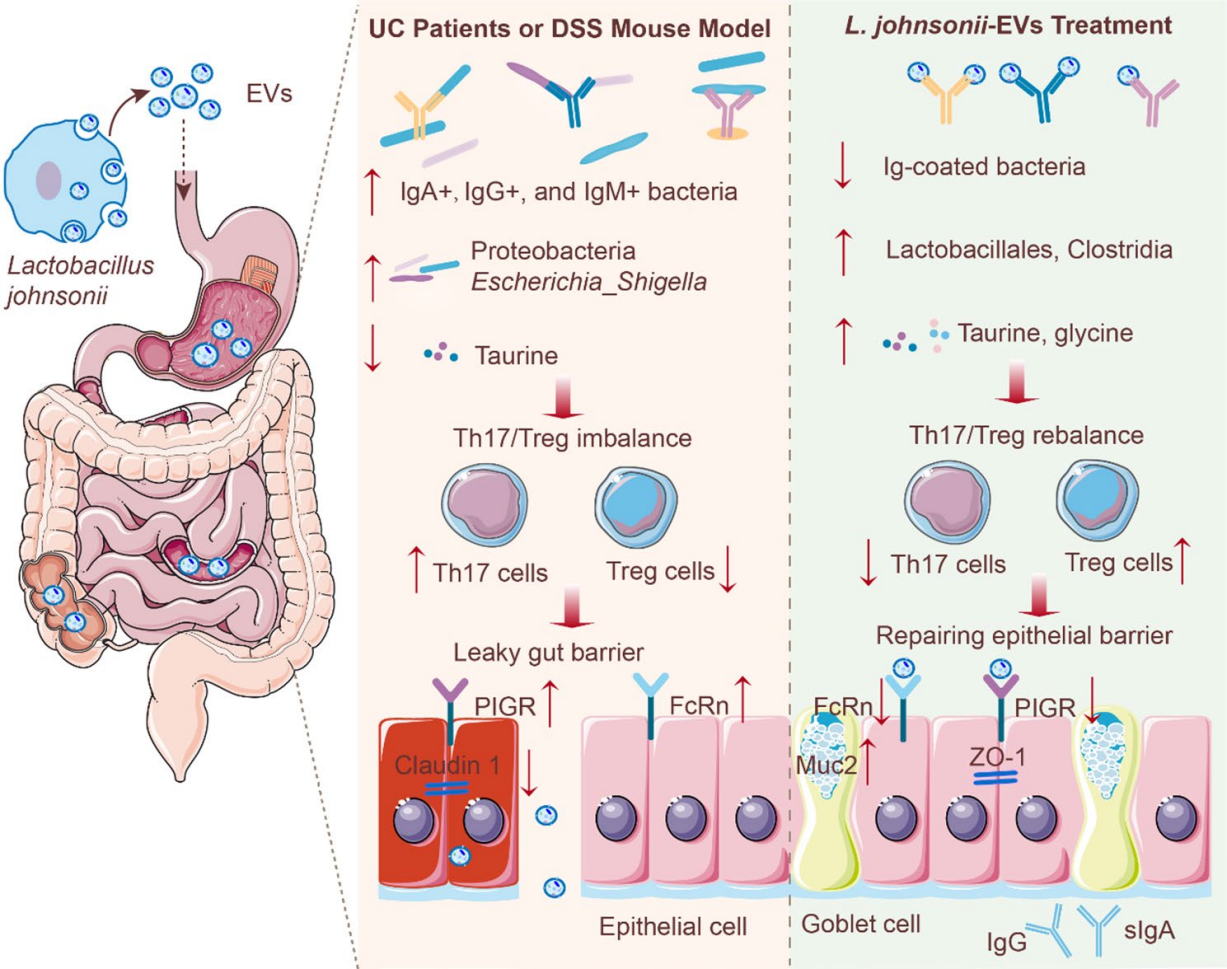

**Supplementary Information:**

The online version contains supplementary material available at 10.1186/s12951-025-03702-6.

## Introduction

Inflammatory bowel disease (IBD), encompassing ulcerative colitis (UC) and Crohn’s disease (CD), is characterized by chronic, relapsing inflammation of the gastrointestinal tract, largely driven by dysregulated immune responses to commensal microbiota [1, 2]. A growing body of evidence implicates immunoglobulin (Ig)-coated bacteria in the initiation and perpetuation of mucosal immune activation, yet the precise mechanisms by which these Ig-tagged microbes contribute to intestinal inflammation remain poorly elucidated [3, 4]. In particular, the dynamic interactions between host immunoglobulins—such as IgA and IgG—and microbial communities have emerged as key modulators of epithelial barrier integrity and immune tolerance, underscoring a critical but underexplored axis in IBD pathogenesis.

Mucosal immunoglobulins—primarily IgA, IgG, and IgM—are central to host–microbiota crosstalk, shaping microbial composition and immune tone at the intestinal barrier [5, 6]. IgA is the predominant mucosal antibody and, under homeostatic conditions, is largely low-affinity and polyreactive, engaging commensals in a non-inflammatory manner to maintain tolerance [7]. In inflammatory settings such as IBD, however, IgA and IgG responses shift to a T cell–dependent program, driven by cognate CD4⁺ T cell–B cell interactions in gut-associated lymphoid tissues, which promote class-switch recombination and affinity maturation [8, 9]. This pathway generates high-avidity IgA that entraps and neutralizes invasive bacteria and IgG that limits systemic dissemination of gut-derived microbes [9–11]. The balance between pro-inflammatory Th17 cells and regulatory T cells (Treg) critically influences the quality of these antibody responses, with Th17 cytokines favoring pathogen-specific high-affinity antibodies, and Treg mediators supporting tolerance-promoting isotypes [8]. IgM, the first isotype produced during humoral responses, contributes to early mucosal defense through broad-spectrum pathogen recognition and polymeric Ig receptor (pIgR)-dependent transport into the lumen [12]. Dysregulation of this coordinated immunoglobulin network in IBD leads to selective coating of pathogenic taxa—particularly by IgA and IgG—amplifying mucosal immune activation and disease severity [13–16].

Beyond direct antibody–microbe interactions, microbe-derived extracellular vesicles (EVs) have emerged as critical agents in modulating host–microbiota communication [17]. These nanosized, membrane-bound vesicles encapsulate bacterial proteins, nucleic acids, and metabolites, facilitating long-range, contact-independent delivery of bioactive cargo to host cells [18, 19]. By influencing both innate and adaptive immune signaling, EVs contribute to mucosal homeostasis and are increasingly recognized as potent effectors of immunoregulation. Notably, EVs secreted by commensal *Lactobacillus* species have demonstrated the capacity to attenuate inflammation and preserve epithelial integrity [20, 21]. Compared with live probiotics, EVs offer superior biostability, safety, and delivery precision—characteristics that make them attractive for microbiota-based therapeutic development. Among these candidates, *Lactobacillus johnsonii* (*L. johnsonii*), a commensal taxon frequently depleted in IBD, has been associated with epithelial barrier maintenance [22], yet its EVs remain largely unexplored in the context of mucosal immune regulation. In light of these unresolved questions, our study investigates whether *L. johnsonii*-derived EVs can modulate immunoglobulin–microbiota interactions and immunometabolic networks in colitis. We hypothesize that these vesicles may offer a novel strategy to reprogram mucosal immune responses and restore intestinal homeostasis by targeting both immune and metabolic axes of inflammation.

Herein, we evaluated *L. johnsonii* and its EVs as therapeutic modulators of IgA/IgG–microbiota interactions, mucosal T cell polarization, and immunometabolic pathways in colitis. Using clinical samples and a dextran sulfate sodium (DSS)-induced mouse model, we show that *L. johnsonii*-derived EVs localize to inflamed colonic tissue, attenuate epithelial injury, suppress pro-inflammatory cytokines, and correct Th17/Treg imbalance. EV administration reduced the abundance of hyper–Ig-coated gut bacteria, downregulated colonic expression of the Ig transporters pIgR and neonatal Fc receptor (FcRn), and remodeled the microbiota toward beneficial taxa. These microbial shifts were accompanied by elevated taurine, a metabolite linked to both epithelial homeostasis and T cell regulation. Taurine supplementation in colitic mice recapitulated key EV-induced effects, including reduced inflammation, barrier restoration, Th17/Treg rebalance, and suppression of pIgR/FcRn expression. To our knowledge, this is the first study to demonstrate that *L. johnsonii*-derived EVs restore mucosal immune balance in colitis by engaging a microbiota–immune–metabolic network involving taurine enrichment, Th17/Treg modulation, and suppression of epithelial Ig transport.

## Methods

### Human sample analysis

Fresh fecal and peripheral blood samples were collected from 33 patients with ulcerative colitis (UC), 21 with Crohn’s disease (CD), and 6 healthy donors at the Department of Gastroenterology, Shenzhen People’s Hospital. Diagnoses of IBD were confirmed via colonoscopy and histopathological assessment. Healthy individuals were recruited in accordance with strict screening criteria described in the fecal microbiota transplantation protocol [23]. Fecal samples were immediately snap-frozen and stored at − 80 °C. Blood samples were centrifuged at 3000 rpm for 10 min at 4 °C, and serum was aliquoted into sterile tubes and stored at − 80 °C for downstream analyses. All sample collection procedures were approved by the institutional ethics committee and conducted in accordance with the Declaration of Helsinki.

### Fecal flow cytometry analysis

The fecal supernatant was washed twice with sterile-filtered PBS or 1% bovine serum albumin before centrifugation at 13,000 rpm for 10 min at 4 °C. Fecal homogenates were then stained with the following antibodies for 30 min: BV421 anti-mouse IgA (BD Biosciences, 743293), PE anti-mouse IgG2b (Biolegend, 406708), and APC anti-mouse IgM (Biolegend, 406509) for mice specimens and APC anti-human IgA (Miltenyi Biotec, 130-116-879), PE anti-human IgG (Biolegend, 398004), and APC anti-human IgM (Biolegend, 314510) for human specimens. The feces solution was then washed twice and resuspended in PBS for flow cytometric analysis by using the BD FACS Canto II cytometer with the logarithmic mode. Data analysis was conducted using FlowJo software (TreeStar, USA). The samples underwent flow cytometry analysis to minimize bacterial autofluorescence and establish forward scatter and side scatter gates. The data pertaining to adherent and non-viable cells underwent processing and gating procedures, with a focus on isolating viable single cells for further analysis. The flow cytometric analysis of IgA-, IgG- or IgG-positive fecal bacteria is non-clustered, detailed gating strategy was based on previous study [16].

### ELISA

Fresh mouse feces samples were washed, suspended in PBS at the concentration of 100 mg/mL, and subjected to serial dilution to determine albumin levels. Human and mouse serum samples were prepared using a three-fold dilution method. The total concentrations of immunoglobulin antibody isotypes in human and mouse serum samples and mouse fecal homogenates were quantified using immunoglobulin-specific ELISA kits (Elabscience, China) in accordance with the manufacturer’s instructions. The samples were incubated at 37 °C for 90 min, followed by incubation with biotinylated anti-human IgA, IgG, or IgE or anti-mouse IgA, IgG, or IgE at 37 °C for 1 h. The plates were read at 450 nm.

### H&E staining

Colon, heart, liver, spleen, lung, and kidney tissues were harvested, snap-frozen in liquid nitrogen, and washed twice with PBS. Tissue segments (~ 0.5 cm) were fixed in 4% formaldehyde, paraffin-embedded, and sectioned at 4 μm. Sections were stained with hematoxylin and eosin (H&E) and evaluated by two blinded investigators.

### Immunohistochemical assay

Both human and mouse colon mucosa specimens were subjected to immunohistochemical (IHC) staining. Myeloperoxidase (MPO, Abcam, ab208670) expression was quantified to determine the extent of acute inflammatory cell infiltration. The expression levels of Claudin 1 (Abcam, ab15098) and Muc2 (Abcam, ab272692) in colon tissue samples were measured to evaluate their association with intestinal barrier function. The semi-quantitative protein grading system was based on two aspects: extent of staining (0 for < 5%, 1 for 6–25%, 2 for 26–50%, 3 for 51–75%, and 4 for 76–100%) and staining intensity (0 for none, 1 for mild, 2 for moderate, and 3 for strong).

### Western blotting

Total protein was extracted using RIPA buffer supplemented with protease and phosphatase inhibitors, and concentrations were determined via bicinchoninic acid assay. Proteins were resolved by SDS‒PAGE and transferred to PVDF membranes. Membranes were probed with primary antibodies against PIGR (Proteintech, 22024-1-AP, 1:1000), FcRn (Abcam, ab228975, 1:1000), or GAPDH (Proteintech, 10494-1-AP, 1:3000) overnight at 4 °C, followed by incubation with HRP-conjugated goat anti-rabbit secondary antibody (Abcam, ab214880) for 30 min at room temperature. Immunoreactive bands were detected using an enhanced chemiluminescence system and quantified with ImageJ. Experiments were performed in triplicate.

### Quantitative reverse transcription-polymerase chain reaction (qRT-PCR)

Total RNA was extracted from the colon tissues of mice, followed by removal of genomic DNA contamination from the total RNA and reverse transcription to cDNA using the PrimeScript™ RT reagent kit (TaKaRa, Japan) in accordance with the manufacturer’s protocol. Subsequently, quantitative PCR was conducted using the qPCR kit (Accurate Biology, China) in 96-well optical plates. The relative cDNA levels were determined using the comparative CT method (2^−ΔΔCt^), with ACTB as the internal reference gene. Pre-designed primers targeting representative inflammatory cytokines, epithelial barrier-related factors, immunoglobulin transporters, and T cell-associated transcription factors were procured from Sangon Biotech; their sequences are Listed in Supplementary Table 1.

### Flow cytometric analysis of mouse colonic T cell subsets

Colonic tissues were enzymatically digested with collagenase IV (0.5%) and DNase I (0.5%) to obtain single-cell suspensions. Cells were stained with fluorochrome-conjugated anti-mouse antibodies against CD45, CD3, CD4, CD25, RORγt, FOXP3, T-bet, and GATA3 (see Supplementary Table 2 for details). Data were acquired on a FACS Canto II cytometer (BD Biosciences) and analyzed with FlowJo software (TreeStar, USA). Nuclear transcription factor gates (T-bet, GATA3, FOXP3, and RORγt) were determined using fluorescence-minus-one controls. Viable single cells were identified after excluding debris and dead cells, and gating strategies followed previously published protocols [24].

### 16 S rRNA profiling and microbiome characterization

Genomic DNA was isolated from human and murine fecal samples using a standardized extraction protocol. The V3-V4 hypervariable regions of the 16 S rRNA gene were amplified and sequenced on an Illumina NovaSeq 6000 platform (Illumina Inc.). Raw sequencing reads were processed using QIIME 2 (v2023.9) for quality filtering, denoising, and chimera removal. High-quality sequences were clustered into operational taxonomic units (OTUs) at 97% similarity using the UPARSE algorithm (v11.0.0, http://www.drive5.com/uparse/). Taxonomic classification of OTUs was performed against the SILVA 138.1 reference database. Downstream statistical analyses, including α-diversity metrics and β-diversity ordination (principal coordinates analysis), were conducted in R (v4.3.1) with the phyloseq and ggplot2 packages.

### Amino acid quantification by liquid chromatography‒mass spectrometry (LC-MS)

Targeted metabolomic analysis of fecal amino acids was performed using UHPLC-MS/MS (ExionLC™ AD coupled with QTRAP 6500+, AB SCIEX) at Novogene Co., Ltd. (Beijing, China). Briefly, 100 mg of fecal samples were homogenized in Liquid nitrogen, diluted 1:10 with water, further diluted to 1:50, and extracted with acetonitrile/methanol (1:1) containing internal standards. After incubation on ice (30 min) and centrifugation (12,000 rpm, 10 min), supernatants were analyzed using an ACQUITY UPLC BEH Amide column (2.1 × 100 mm, 1.7 μm) at 50 °C. The mobile phase consisted of 0.1% formic acid in 5 mM ammonium acetate (A) and acetonitrile (B) with a gradient elution. Quantification was conducted in positive MRM mode. Calibration curves were established using standard solutions, with correlation coefficients (r) > 0.99. The limit of quantification was defined at a signal-to-noise ratio (S/N) of 10. The Linear regression equations and quantitative results of 23 amino acids in fecal samples were obtained. Subsequently, Pearson correlation heatmaps were constructed to analyze the relationships between the differential microbiota identified by LEfSe analysis from murine fecal 16 S rRNA sequencing and the levels of 23 targeted amino acids in each group. After identifying the key metabolites, KEGG pathway enrichment analysis was performed for the selected metabolites.

### L. johnsonii strain culture

The detailed procedures for the isolation and purification of *L. johnsonii* (ATCC 33200) from healthy donors have been covered in our previous studies [24–26], and its nucleotide sequence information is provided in Supplementary Table 3. *L. johnsonii* was cultured in MRS broth at 37 °C for 48 h under anaerobic conditions. For oral gavage, *L. johnsonii* was harvested at the logarithmic phase by centrifugation, washed, and resuspended in PBS to reach a density of 1*10^9^ colony forming units (CFUs)/mL.

### Isolation and purification of L. johnsonii-EVs

Under anaerobic conditions, a single colony of *L. johnsonii* was isolated from an MRS agar plate following a 24-hour incubation at 37 °C and subsequently inoculated into MRS broth. The culture was incubated at 37 °C for 48 h to facilitate bacterial growth. To ensure optimal bacterial density, the culture was refreshed by a 1:100 dilution in fresh MRS broth and incubated at 37 °C for an additional 24 h until the optical density at 600 nm (OD600) reached 1.0. After incubation, the bacterial suspension was centrifuged at 5,000 × g for 10 min to pellet the bacterial cells. The harvested bacteria were resuspended in sterile phosphate-buffered saline (PBS) at a concentration of 0.2 g/mL and homogenized using an ultrasonic homogenizer on ice. The homogenization process consisted of 20 min of sonication at 65 W, with a cycle of 3-second pulses and 5-second intervals to ensure efficient cell lysis while minimizing EVs degradation. The resulting suspension was subjected to centrifugation at 10,000 × g for 20 min at 4 °C to remove cellular debris. The supernatant was carefully collected and filtered through a 0.22 μm membrane filter to eliminate residual intact bacteria and particulate matter. The filtered EVs-containing solution was concentrated using a 100 kDa ultrafiltration unit (Millipore). To achieve high-purity EVs, the concentrated solution was ultracentrifuged at 150,000 × g for 2 h at 4 °C using an Optima™ XE-100 ultracentrifuge equipped with a Type 70 Ti rotor (Beckmann Coulter, USA) [20]. The purified EVs were resuspended in 1x PBS (pH 7.4) and stored at −80 °C until further use.

### Characterization of L. johnsonii-EVs

The protein concentration of the purified EVs was quantified using a bicinchoninic acid (BCA) protein assay kit (EpiZyme) according to the manufacturer’s instructions. The morphology of the EVs was visualized using transmission electron microscopy (TEM) (HT7700 electron microscope, Hitachi). Additionally, the size distribution, hydrodynamic diameter, and zeta potential of the EVs were analyzed using dynamic light scattering (DLS) (Zetasizer Nano AS90, Malvern Panalytical) as described in our previous study. This comprehensive protocol ensures the isolation and characterization of high-purity *L. johnsonii*-derived EVs, enabling their downstream application in experimental colitis models and mechanistic studies [20].

### Development of an acute DSS colitis mice model

Six- to 8-week-old male BALB/c mice, bred under pathogen-free conditions, were procured from the Guangdong Medical Laboratory Animal Center (SCXK 2018-0002, China). These mice were maintained in a controlled environment designed to meet specific pathogen-free standards. Ethics approval for the animal research protocols was granted by the Ethics Committee of Shenzhen People’s Hospital. The care and use of animals in this study were conducted in compliance with the guidelines of the Institutional Animal Care and Use Committee.

### Efficacy of L. johnsonii-EVs in DSS-induced colitis

Acute colitis was induced in BALB/c mice by administering 3% (w/v) DSS (Sigma, USA) in drinking water ad Libitum for 7 consecutive days, as previously described [20, 24]. Mice were randomly assigned to four groups (*n* = 5 per group): Healthy control (HC), DSS + PBS (PBS), DSS + *L. johnsonii* (*Lj*), and DSS + *L. johnsonii*-derived EVs (EVs). Mice in the treatment groups received daily oral gavage of either PBS, live *L. johnsonii* (100 µL/10 g body weight), or EVs (2.5 mg/mL, 100 µL/10 g) throughout the DSS administration period [27]. Control animals received plain water and equivalent PBS gavage. Body weight, stool consistency, and rectal bleeding were recorded daily to calculate the disease activity index (DAI). At endpoint, mice were euthanized via intraperitoneal injection of tribromoethanol (1.25%, 200 µL/10 g). Colon tissues and fecal samples were collected for further analyses. Histopathological scoring (maximum score = 9), based on epithelial injury, crypt architecture, and inflammatory infiltration, was independently evaluated by two blinded investigators [24].

### Fluorescent labeling of L. johnsonii-EVs

For fluorescent tracking, *L. johnsonii*-EVs were labeled with the lipophilic dye DiI (Thermo Fisher Scientific). Briefly, 5 µL of DiI (1 mM) was added to 1 mL of EVs suspension, thoroughly mixed, and incubated for 0.5 h at 37 °C. Unbound dye was removed by purification using a 100 kDa ultrafiltration centrifugal filter (Millipore). The labeled EVs were resuspended in PBS and stored at 4 °C for subsequent experiments.

### Stability assessment of L. johnsonii-EVs

The stability of *L. johnsonii*-derived EVs was evaluated under simulated gastrointestinal conditions. Briefly, *L. johnsonii*-derived EVs were incubated in simulated gastric fluid (SGF, pepsin, pH 1.5) or simulated intestinal fluid (SIF, trypsin, pH 6.8) at 37 °C for 30 min. After incubation, the EVs were isolated using exosome spin columns with a molecular weight cutoff of 4,000 (MW4000) to ensure purity and stability. The morphological integrity of the EVs after treatment was visualized by TEM. This experimental procedure was modified based on our previous methodology to mimic physiologically relevant conditions and evaluate the structural stability of bacterial EVs [20].

### Tissue-specific distribution of L. johnsonii-EVs

To evaluate the gastrointestinal and systemic biodistribution of *L. johnsonii*-EVs, colitis-induced and healthy control mice received a single oral dose of 200 µL PBS containing DiI-labeled EVs (2.5 mg/mL). At 4, 12, 24 h and 48 h post-administration, mice were euthanized via CO₂ asphyxiation. Gastrointestinal tract tissues (stomach, small intestine, colon) and systemic organs (brain, heart, liver, spleen, lung, kidney) were excised and immediately subjected to fluorescence imaging using an IVIS Spectrum system (PerkinElmer, Hopkinton, USA). Signal intensity was quantified to determine DiI-EVs accumulation across tissues, enabling spatiotemporal tracking of EVs dissemination.

### Colonic uptake of L. johnsonii-EVs

To examine the colonic uptake of *L. johnsonii* and its extracellular vesicles (EVs), healthy mice and colitis-induced mice were fasted for 24 h and subsequently administered 200 µL of DiI-labeled EVs (2.5 mg/mL) or 100 µM FITC-D-lysine-labeled *L. johnsonii* via oral gavage. Six hours post-administration, colon tissues were harvested and fixed overnight in 4% paraformaldehyde at 4 °C. Tissues were cryoprotected in 30% sucrose-PBS at 4 °C for 48 h, embedded in optimal cutting temperature compound (OCT), and frozen. Serial Sect. (5-µm thickness) were prepared using a cryostat. Sections were washed with PBS, blocked with 3% bovine serum albumin (BSA) to reduce nonspecific binding, and counterstained with 4′,6-diamidino-2-phenylindole (DAPI; Beyotime Biotechnology). Fluorescence imaging was performed using a high-resolution scanner (Pannoramic MIDI, 3DHISTECH, Hungary) to visualize EVs and bacterial internalization.

### Systemic biosafety assessment of L. johnsonii-EVs

To evaluate the systemic safety of *L. johnsonii*-EVs, blood samples were collected from colitis-induced mice administered *L. johnsonii*-EVs (100 µL/10 g/day) for 7 consecutive days. Serum levels of creatine kinase-MB (CK-MB), alanine aminotransferase (ALT), aspartate aminotransferase (AST), creatinine (Crea), and urea were quantified using standardized enzymatic assays. Healthy mice and untreated colitis mice served as controls. Major organs (heart, liver, spleen, lung, kidney) were harvested, fixed in 4% paraformaldehyde, paraffin-embedded, and sectioned at 4 μm thickness for H&E staining. Histopathological analysis was conducted by two blinded investigators to assess tissue toxicity.

### Transcriptomic data acquisition and analysis

The GSE14580 dataset was retrieved from the Gene Expression Omnibus (GEO) repository (https://www.ncbi.nlm.nih.gov/geo/). Raw MINiML-formatted files were preprocessed following the standardized pipeline described in the dataset metadata. Differential expression of PIGR and FcRn in colonic mucosal samples was analyzed using R v4.0.3 (R Foundation for Statistical Computing) with the limma package for microarray normalization and statistical modeling. Significance thresholds were defined as the *p* < 0.05.

### Efficacy of taurine in DSS-induced colitis

An acute colitis model was established in BALB/c mice via 7-day administration of 3% (w/v) DSS, followed by allocation into three groups (*n* = 5 per group): HC, DSS + PBS, and DSS + taurine. Taurine-treated mice received daily oral gavage of 0.5% (w/v) taurine (Macklin, T6017; 50 µL/10 g body weight) during DSS administration, while controls received PBS. Subsequent procedures for clinical assessment, tissue collection, and histopathological scoring followed the protocols described above.

### Statistical analysis

Data analysis was performed using IBM SPSS Statistics v24.0 (IBM Corp.). All experiments were independently replicated at least three times. Normally distributed continuous variables are presented as mean ± standard deviation (SD). Intergroup differences were determined using a two-tailed unpaired Student’s t-test. Parametric correlations between variables were assessed via Pearson’s correlation coefficient. Statistical significance was defined as *p* < 0.05, with thresholds denoted as follows: *p* < 0.05 (*), *p* < 0.01 (**), *p* < 0.001 (***); ns, nonsignificant.

## Results

### Correlation analysis between immunoglobulin-coated gut microbial consortia and IBD activity

**Fig. 1:**
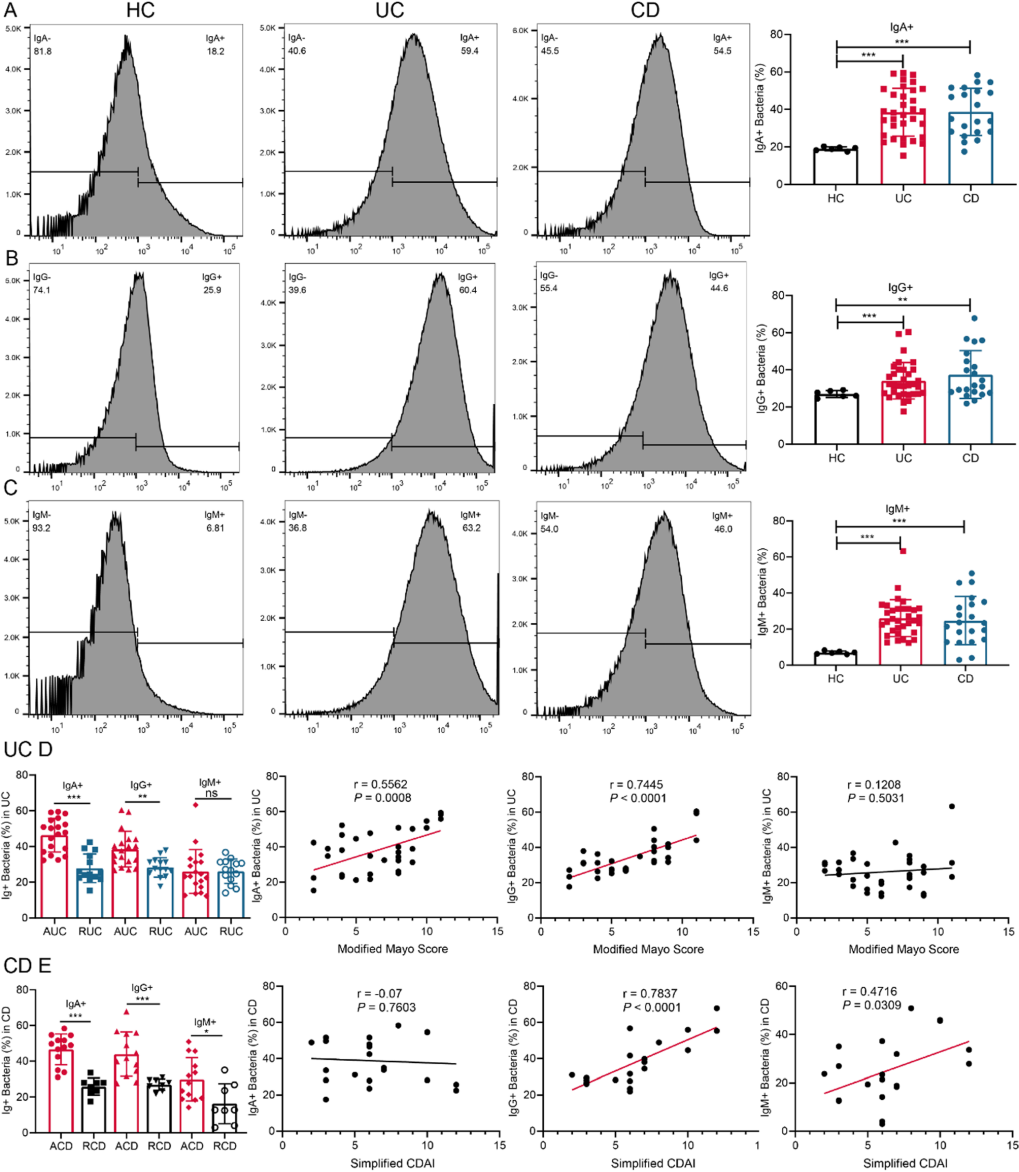
Correlation analysis between immunoglobulin-coated gut microbial consortia and IBD activity. (**A-C**) Bacterial flow cytometry images and quantitative analysis showing IgA-, IgG-, or IgM-bound bacteria in fecal samples from UC (*n* = 33), CD (*n* = 21), and HC (*n* = 6). (**D**) Percentage of IgA^+^, IgG^+^, or IgM^+^ coated fecal bacteria in active (*n* = 19) and inactive (*n* = 14) UC, and correlation with UC activity (Modified Mayo Score). (**E**) Percentage of IgA^+^, IgG^+^, or IgM^+^ coated fecal bacteria in active (*n* = 13) and inactive (*n* = 8) CD, and correlation with CD activity (Simplified CDAI). Data are presented as mean ± SD. *p < 0.05, **p < 0.01, ***p < 0.001. Abbreviations: Simplified CDAI, Simplified Crohn’s Disease Activity Index

We performed a medium-scale analysis of immunoglobulin (Ig) coating on gut microbial consortia in patients with inflammatory bowel disease (IBD), including 33 patients with ulcerative colitis (UC; 19 active, 14 inactive), 21 with Crohn’s disease (CD; 13 active, 8 inactive), and 6 healthy controls. Clinical characteristics such as body mass index, Montreal classification, disease location, inflammatory burden, infection status, and medication history were assessed (Table 1). Using bacterial fluorescence-activated cell sorting, we quantified fecal bacteria coated with IgA, IgG, and IgM. Compared with healthy controls, both UC and CD patients exhibited significantly increased proportions of highly IgA-coated (IgA^+^), IgG-coated (IgG^+^), and IgM-coated (IgM^+^) bacteria (Fig. 1A–C), indicating enhanced immune targeting of gut microbiota in IBD. Stratification by disease activity revealed higher levels of IgA^+^ and IgG^+^ bacteria in active UC (AUC) than in UC in remission (RUC) (Fig. 1D). Furthermore, the abundance of IgA^+^ and IgG^+^ bacteria positively correlated with UC activity assessed by the modified Mayo score (IgA^+^: *r* = 0.5562, *p* = 0.0008; IgG^+^: *r* = 0.7445, *p* < 0.0001), whereas IgM^+^ bacteria showed no significant correlation. Similarly, active CD (ACD) patients displayed increased proportions of IgA^+^, IgG^+^, and IgM^+^ bacteria compared with CD in remission (RCD) (Fig. 1E). Notably, IgG^+^ bacteria strongly correlated with CD activity measured by the simplified CDAI (*r* = 0.7837, *p* < 0.0001), and IgM^+^ bacteria showed a moderate correlation (*r* = 0.4716, *p* = 0.0309), while IgA^+^ bacteria showed no significant association. Collectively, these findings demonstrate that patients with active IBD harbor higher levels of immunoglobulin-coated gut bacteria, particularly IgA-and IgG-bound consortia, which strongly correlate with disease activity.

**Table 1:** Clinical characteristics of IBD patients

Variables	Total patients N = 54
Age, Mean (Range)	38.90 (17–77)
Gender, N (%)	
Female	21 (38.89)
Male	33 (61.11)
BMI, Mean (Range)	20.20 (15.35–25.95)
CMV infection, N (%)	5 (9.25)
*C. difficile* infection, N (%)	11 (20.37)
The Montreal Classification, UC, N (%)	33
E1, proctitis	4 (12.12)
E2, left sided	18 (54.55)
E3, pancolitis	11 (33.33)
The Montreal Classification, CD, N (%)	21
Location	
L1, ileal	1 (4.76)
L2, colonic	3 (14.29)
L3, ileocolonic	17 (80.95)
L4, upper GI tract	4 (19.05)
Behaviour	
B1, non-stricturing, non-penetrating	9 (42.86)
B2, stricturing	10 (47.62)
B3, penetrating	2 (9.52)
P, perianal	7 (33.33)
Treatments, N (%)	
Aminosalicylic acid	50 (92.59)
Corticosteroids	17 (31.48)
Biologics	16 (29.63)
Vedolizumab (blocks integrin α4β7)	9 (16.67)
Infliximab, adalimumab (anti-TNFα)	7 (12.96)
Antibiotics (metronidazole and moxifloxacin)	9 (16.67)
Probiotics	17 (31.48)
FMT	10 (18.52)

CMV, cytomegalovirus infection; C. difficile, Clostridioides difficile

### Inflammatory characteristics of active IBD

**Fig. 2:**
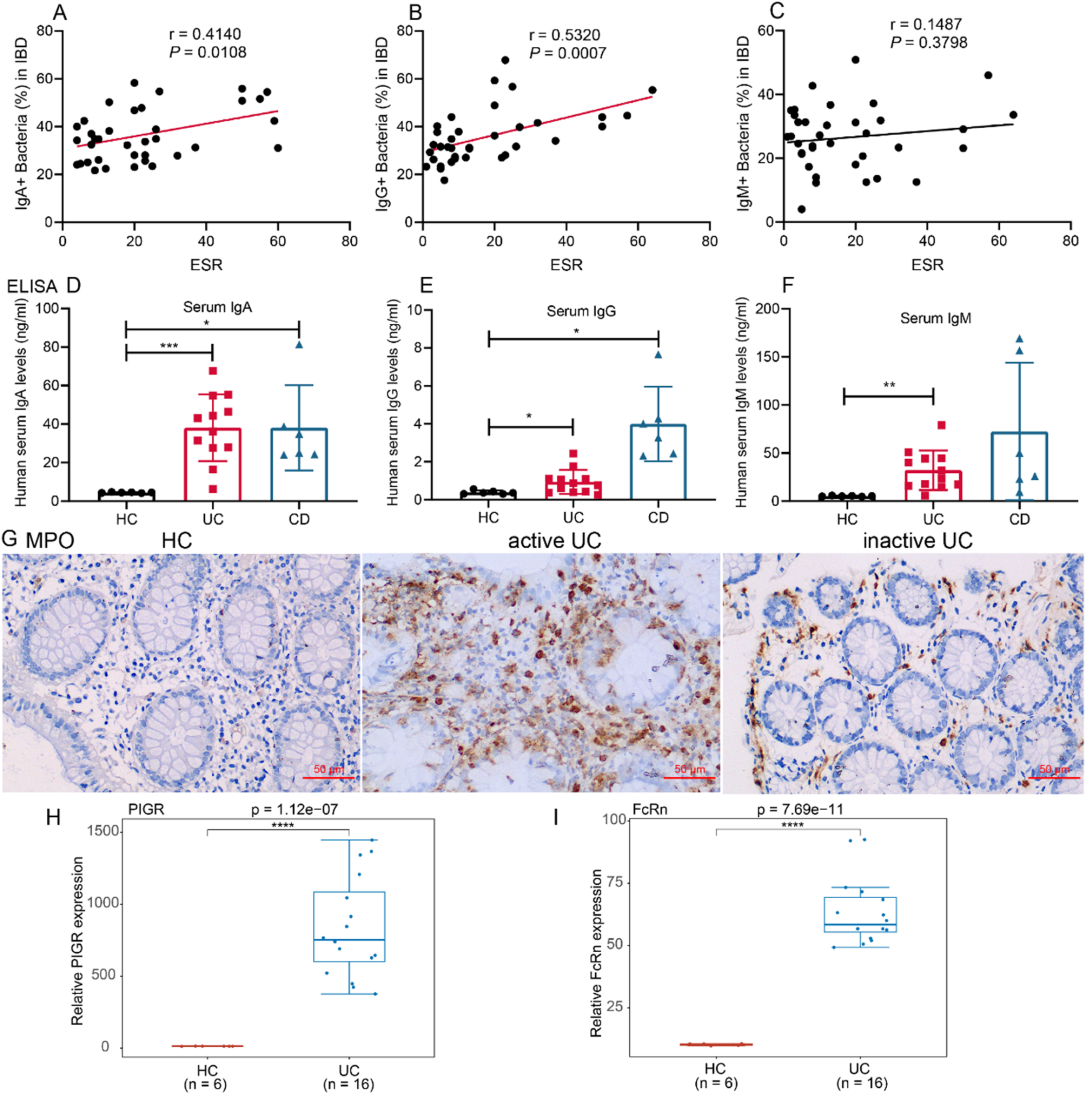
Inflammatory characteristics of active IBD. (**A-C**) Correlation analysis between IgA^+^, IgG^+^, or IgM^+^ coated fecal bacteria and ESR (*n* = 37). (**D-F**) ELISA results of serum IgA, IgG or IgM levels in IBD patients (UC, *n* = 12; CD, *n* = 6; HC, *n* = 6). (**G**) Representative IHC images of MPO in UC colon tissues (×200). Box and dot plot of PIGR (**H**) or FcRn (**I**) gene expression in active UC (*n* = 16) and HC (*n* = 6) colon tissues from the GSE14580 dataset. Data are presented as mean ± SD. *p < 0.05, **p < 0.01, ***p < 0.001. Abbreviations: ESR, erythrocyte sedimentation rate; MPO, myeloperoxidase

Erythrocyte sedimentation rate (ESR) is a key clinical marker for assessing IBD activity. We evaluated the association between ESR levels and immunoglobulin-coated (Ig^+^) fecal microbiota in IBD patients. A moderate positive correlation was identified between ESR and highly IgA-coated bacteria (*r* = 0.4140, *p* = 0.0108; Fig. 2A), as well as between ESR and highly IgG-coated bacteria (*r* = 0.5320, *p* = 0.0007; Fig. 2B). In contrast, no significant correlation was observed between ESR and highly IgM-coated bacteria (Fig. 2C). We further assessed serum immunoglobulin levels in patients with active IBD. Serum concentrations of IgA, IgG, and IgM were significantly elevated in active UC patients compared to healthy controls (Fig. 2D–F). In active CD patients, only serum IgA and IgG levels were markedly increased relative to controls (Fig. 2D–F). To evaluate mucosal inflammation, IHC staining for MPO was performed, revealing substantially reduced neutrophil infiltration in the colonic tissue of patients with inactive UC compared to those with active disease (Fig. 2G). Additionally, analysis of PIGR and FcRn gene expression in colonic tissues from active UC patients (*n* = 16) and healthy controls (*n* = 6) using the GSE14580 dataset demonstrated significantly higher RNA expression levels of both genes in active UC (*p* < 0.001; Fig. 2H–I).

### L. johnsonii depletion in UC and characterization of its derived EVs

**Fig. 3:**
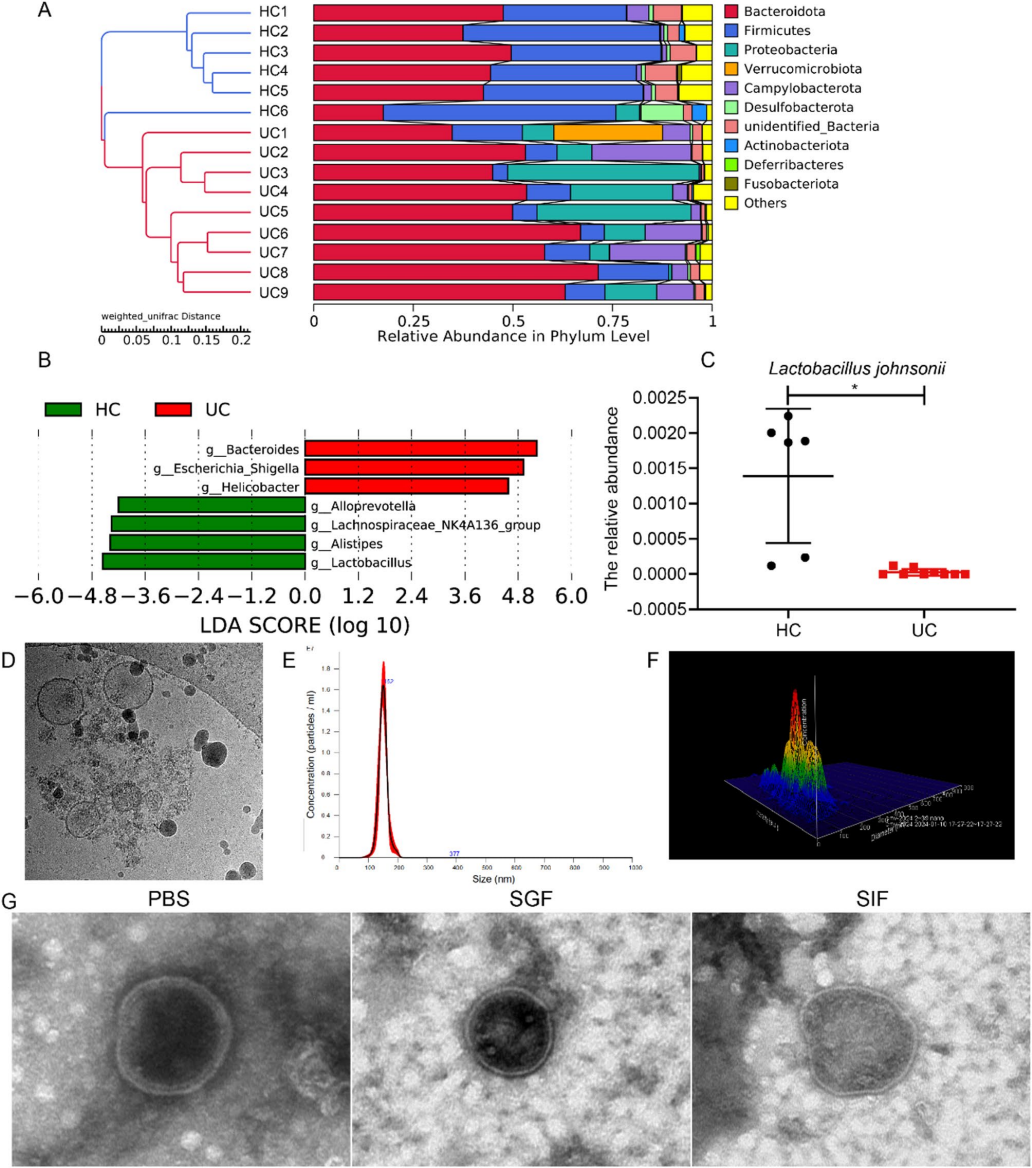
*L. johnsonii* depletion in UC and characterization of its derived EVs. (**A**) 16 S rRNA sequencing of gut microbiota in UC patients (*n* = 9) vs. healthy controls (HC, *n* = 6). (**B**) LEfSe-based LDA analysis showing reduced *Lactobacillus* abundance in active UC. (**C**) Decreased *L. johnsonii* abundance in active UC compared to HC. (**D**) TEM analysis of *L. johnsonii*-derived EVs. (**E**) EVs’ diameter ranges from 100 nm to 200 nm, with a median of 152 nm. (**F**) 3D surface plot of EVs. (**G**) TEM analysis of *L. johnsonii*-derived EVs in PBS, SGF and SIF (×20000). Data are presented as mean ± SD. *p < 0.05

To explore gut microbiota alterations in UC, fecal samples from 9 patients with active UC and 6 healthy controls were analyzed by 16 S rRNA gene sequencing. β-diversity analysis based on weighted UniFrac distances, followed by unweighted pair group method with arithmetic mean (UPGMA) clustering, demonstrated a distinct separation between UC patients and healthy controls, indicating marked gut microbial dysbiosis in UC. Taxonomic profiling revealed a significant reduction in Firmicutes and enrichment of Proteobacteria in UC patients compared with controls (Fig. 3A). Linear discriminant analysis Effect Size (LEfSe) analysis further identified *Lactobacillus* depletion and enrichment of *Helicobacter* and *Escherichia*_*Shigella* in the UC gut microbiota (Fig. 3B). Notably, the relative abundance of *L. johnsonii* was significantly decreased in UC patients (Fig. 3C), suggesting its potential relevance in UC pathogenesis and therapy.

Subsequently, *L. johnsonii* was cultured and its EVs were isolated by ultrasonication, ultracentrifugation, and ultrafiltration. TEM analysis revealed that *L. johnsonii*-derived EVs exhibited a typical spherical morphology with an intact lipid bilayer structure (Fig. 3D). Nanoparticle tracking analysis showed that EVs sizes ranged from 100 to 200 nm, with a median diameter of 152 nm (Fig. 3E). A 3D surface plot based on particle diameter and concentration further visualized the particle size distribution and uniformity of *L. johnsonii*-EVs (Fig. 3F). Additionally, zeta potential analysis revealed a negative surface charge of −0.31 mV, indicating their stable colloidal properties in suspension (Supplementary Fig. S1). To evaluate their stability in gastrointestinal environments, *L. johnsonii*-EVs were incubated in SGF and SIF at 37 °C for 30 min. TEM observations confirmed that EVs maintained their structural integrity and exhibited minimal size variation compared with PBS incubation (Fig. 3G). These findings highlight the robust structural stability of *L. johnsonii*-derived EVs under harsh gastrointestinal conditions, supporting their potential as a stable and functional candidate for oral therapeutic delivery in UC.

### Tracking biodistribution and epithelial association of L. johnsonii-derived EVs in vivo

**Fig. 4:**
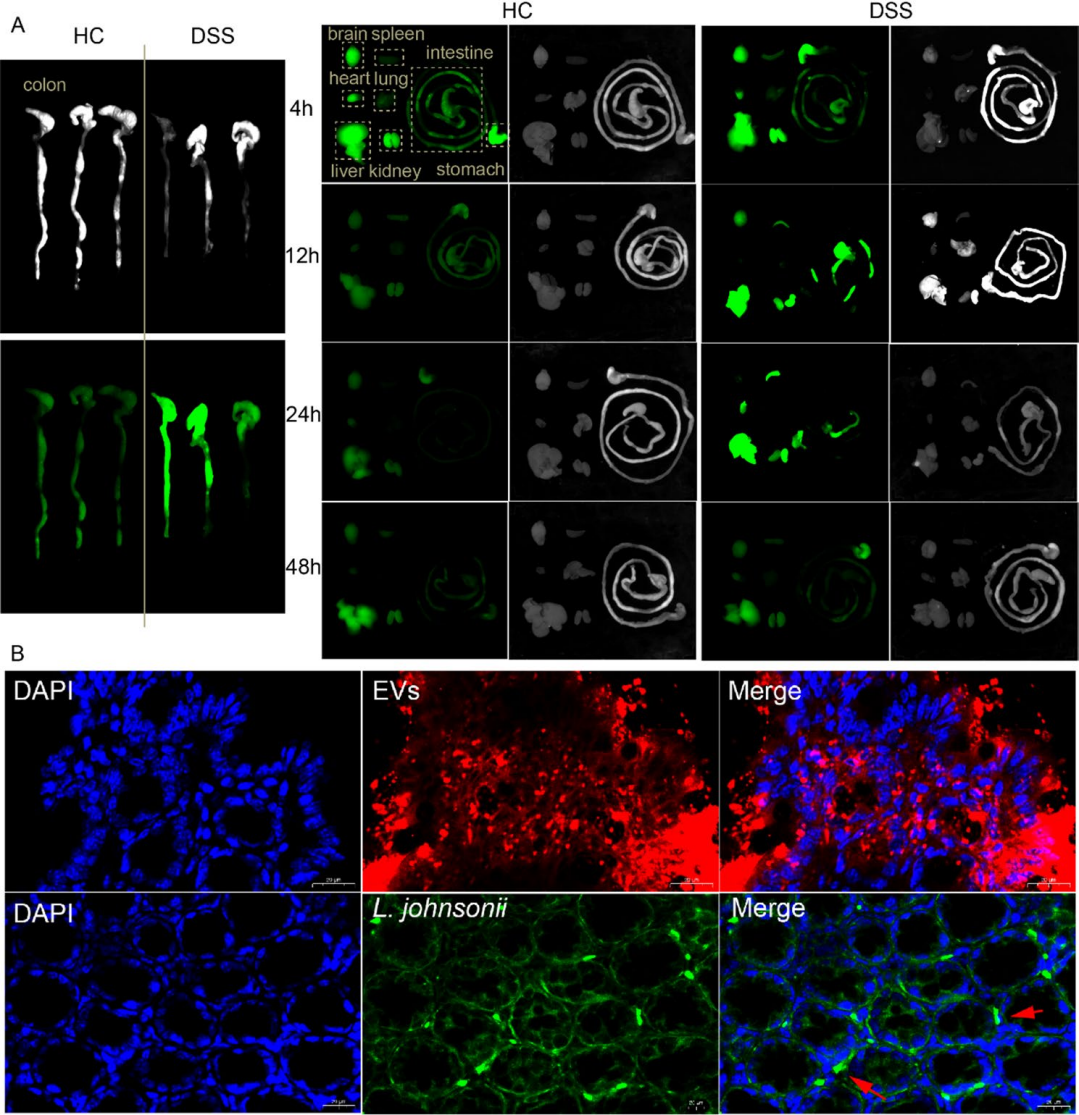
Tracking biodistribution and epithelial association of *L. johnsonii*-derived EVs in vivo. (**A**) In vivo imaging showing the dynamic distribution of DiI-labeled *L. johnsonii*-derived EVs in the heart, brain, Liver, spleen, lung, kidney, stomach, and intestine of DSS-induced colitis mice at 4, 12, 24, and 48 h post-administration. (**B**) Confocal microscopy images 6 h post-gavage showing colonic distribution of DiI-labeled EVs (red) and FITC-d-Lys-labeled *L. johnsonii* (green) in DSS mice (×500). EVs were closely associated with the colonic epithelium, whereas *L. johnsonii* primarily localized within the crypt mesenchyme

To investigate the in vivo biodistribution of *L. johnsonii*-derived EVs following oral administration, DiI-labeled EVs were gavaged to DSS-induced colitis mice and healthy controls. Whole-body and organ fluorescence imaging was performed at 4, 12, 24, and 48 h post-administration. In DSS mice, EV-associated fluorescence was detectable in the colon as early as 4 h, became visibly stronger and more extensive by 12 h, and then gradually declined by 48 h (Fig. 4A). In healthy mice, colonic fluorescence remained relatively weak throughout the observation period, indicating inflammation-associated relative enrichment of EVs in the colon of DSS mice. In both groups, transient fluorescence signals were observed in major organs including the heart, brain, Liver, lung, and kidney within 12 h post-administration, but these diminished over time, consistent with physiological clearance (Fig. 4A). To further examine mucosal localization, UC mice were fasted for 24 h prior to oral gavage with FITC-d-Lys-labeled *L. johnsonii* and DiI-labeled EVs. Confocal microscopy of colonic sections at 6 h post-gavage showed that *L. johnsonii* predominantly localized within the crypt mesenchyme without evident epithelial interaction, whereas EVs were clearly observed along the colonic epithelium in DSS mice (Fig. 4B). These findings demonstrate that *L. johnsonii*-derived EVs display visible enrichment in inflamed colonic tissue and establish close association with the epithelial surface in vivo, supporting their potential as inflammation-responsive delivery vehicles for gut-targeted applications.

### Biological safety of L. johnsonii-derived EVs in mice

**Fig. 5:**
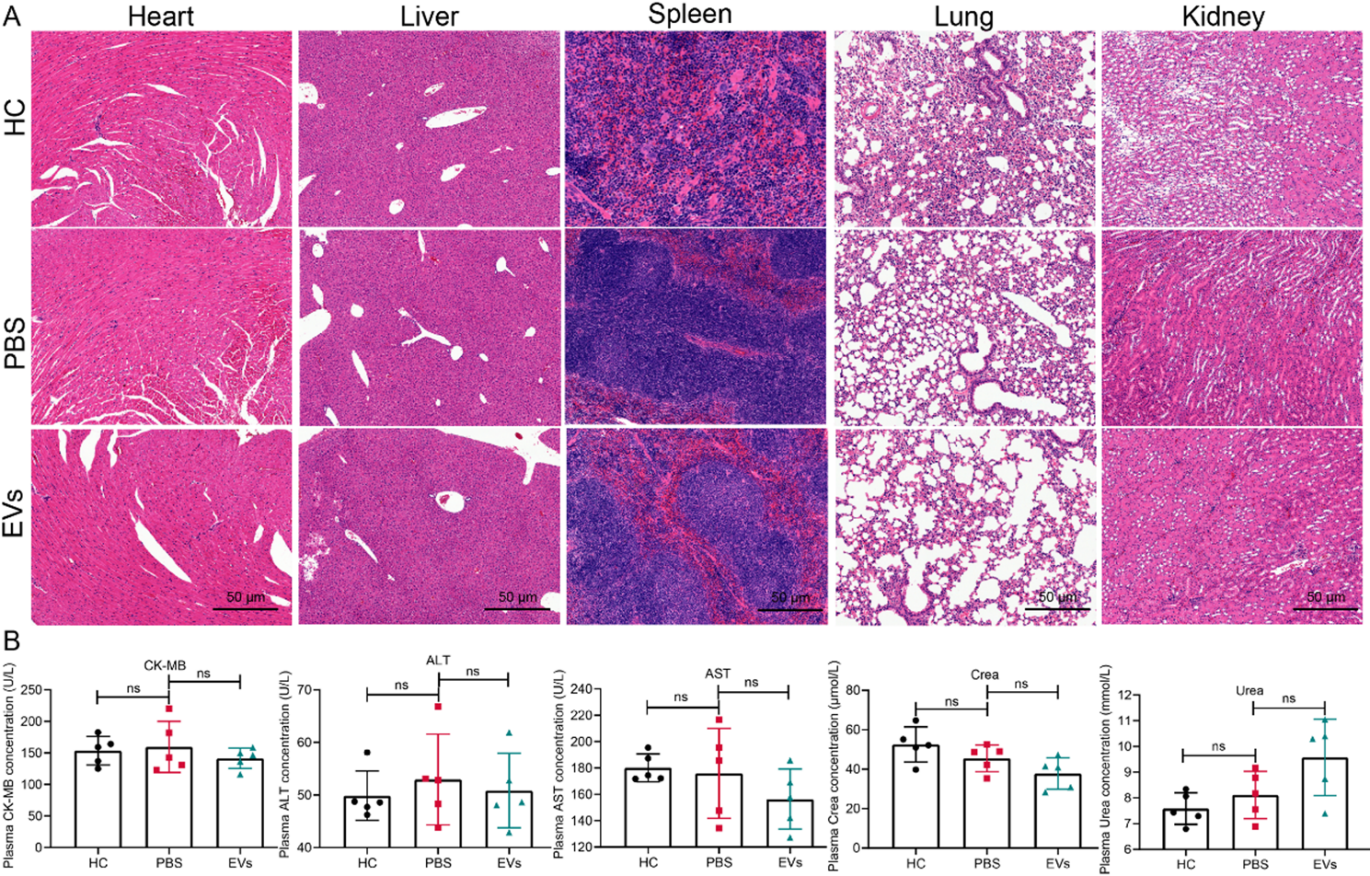
Biological safety of *L. johnsonii*-derived EVs in mice. (**A**) H&E staining showed no pathological changes in the heart, liver, spleen, lung, or kidney. (**B**) Biochemical analysis showed no abnormalities in plasma CK-MB, ALT, AST, creatinine (Crea), or urea levels. ns, not significant

To evaluate the biological safety of *L. johnsonii*-derived EVs, UC mice were orally administered with EVs, followed by histological and biochemical analyses. H&E staining revealed no observable pathological changes in major organs, including the heart, liver, spleen, lung, and kidney, indicating no evident tissue toxicity after EVs treatment (Fig. 5A). Furthermore, plasma biochemical parameters were measured to assess systemic toxicity. The levels of CK-MB, ALT, AST, Crea, and urea remained within normal ranges and showed no significant differences compared with control mice, suggesting no hepatic, renal, or cardiac dysfunction (Fig. 5B). These results demonstrate that *L. johnsonii*-derived EVs possess favorable in vivo biocompatibility and do not induce systemic toxicity in mice, supporting their potential for therapeutic applications.

### Both L. johnsonii and its derived EVs alleviated DSS-induced colitis and enhanced the gut barrier

**Fig. 6:**
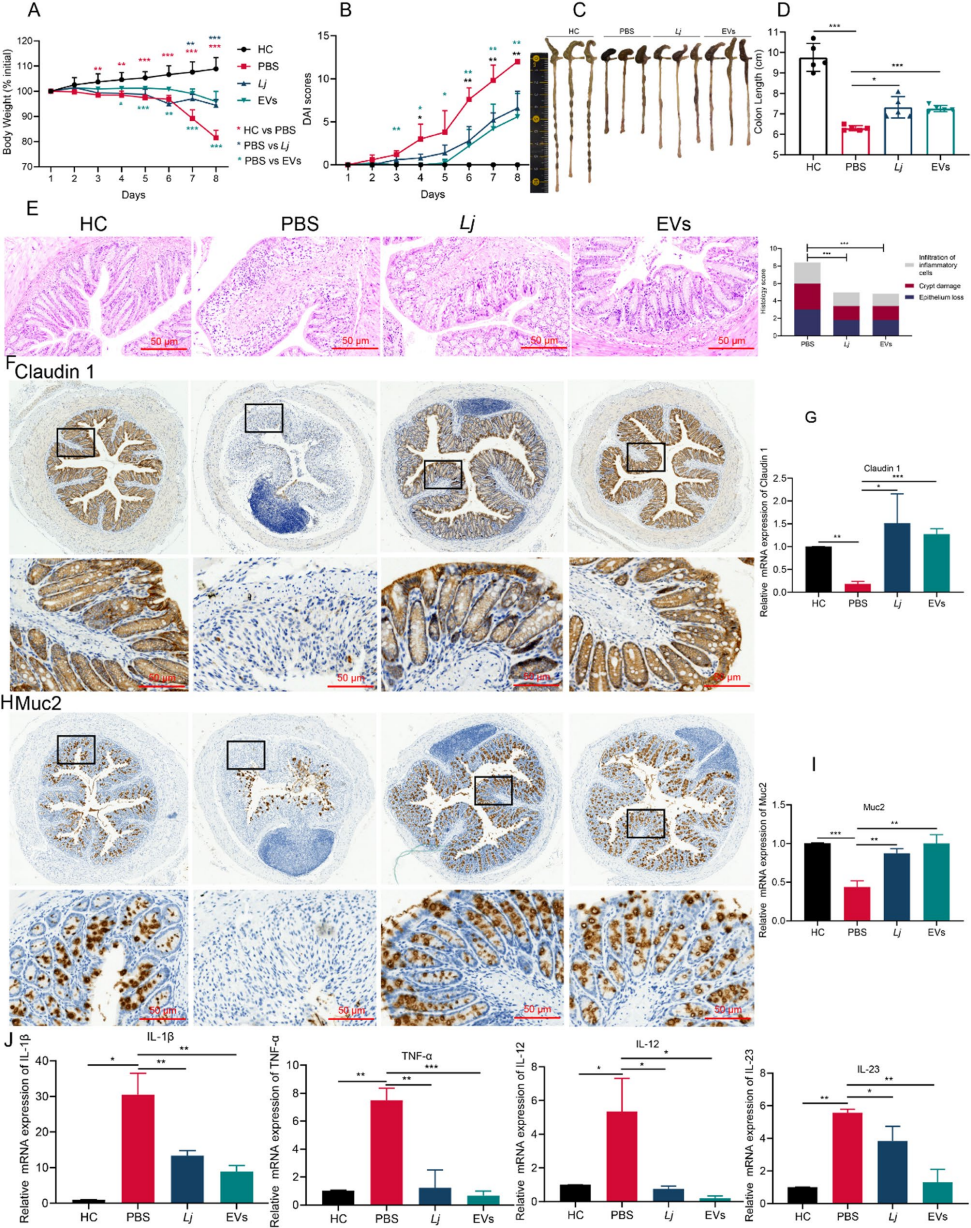
Both *L. johnsonii* and its derived EVs alleviated DSS-induced colitis and enhanced the gut barrier. (**A**) Body weight loss (*n* = 5). (**B**) DAI scores. (**C**) Representative images of the colon. (**D**) Colon length (*n* = 5). (**E**) H&E staining (×200) and pathological score of rectum. (**F-G**) IHC images and partial enlargement of Claudin 1 (×200), and its mRNA expression. (**H-I**) IHC images and partial enlargement of Muc2 (×200), and mRNA expression of Muc2. (**J**) mRNA expression of inflammatory markers (*n* = 3). Data are presented as mean ± SD. *p < 0.05, **p < 0.01, ***p < 0.001. Abbreviations: DSS, dextran sulfate sodium

Following the confirmation of the in vivo safety profile of *L. johnsonii*-derived EVs, we next investigated their therapeutic efficacy in a DSS-induced acute colitis mouse model. Considering the well-established influence of gut microbiota on intestinal homeostasis, we selected both *L. johnsonii* (*Lj*) and its EVs as microbiome-based interventions to alleviate colitis symptoms and restore gut barrier function. Mice receiving *Lj* or EVs exhibited significantly improved clinical indicators compared with PBS-treated controls, including reduced weight loss, lower DAI, and increased colon length (Fig. 6A–D). Histopathological examination demonstrated that both treatments effectively mitigated mucosal damage, reduced neutrophil infiltration, and preserved colonic architecture (Fig. 6E). To evaluate their effect on epithelial barrier integrity, IHC analysis revealed upregulated expression of Claudin 1 and Muc2 in the colons of *Lj*- and EVs-treated mice (Fig. 6F, H), which was further confirmed by qPCR (Fig. 6G, I). Additionally, pro-inflammatory cytokines such as IL-1β, TNF-α, IL-12, and IL-23 were significantly downregulated in EVs- and *Lj*-treated groups compared with PBS controls (Fig. 6J). Together, these findings demonstrate that both *Lj* and its EVs not only exhibit excellent biocompatibility but also exert protective effects against intestinal inflammation by restoring epithelial integrity and modulating immune responses, underscoring their promise as safe and effective microbiome-based therapeutics for colitis.

### Both L. johnsonii and its derived EVs restored the Th17/Treg balance

**Fig. 7:**
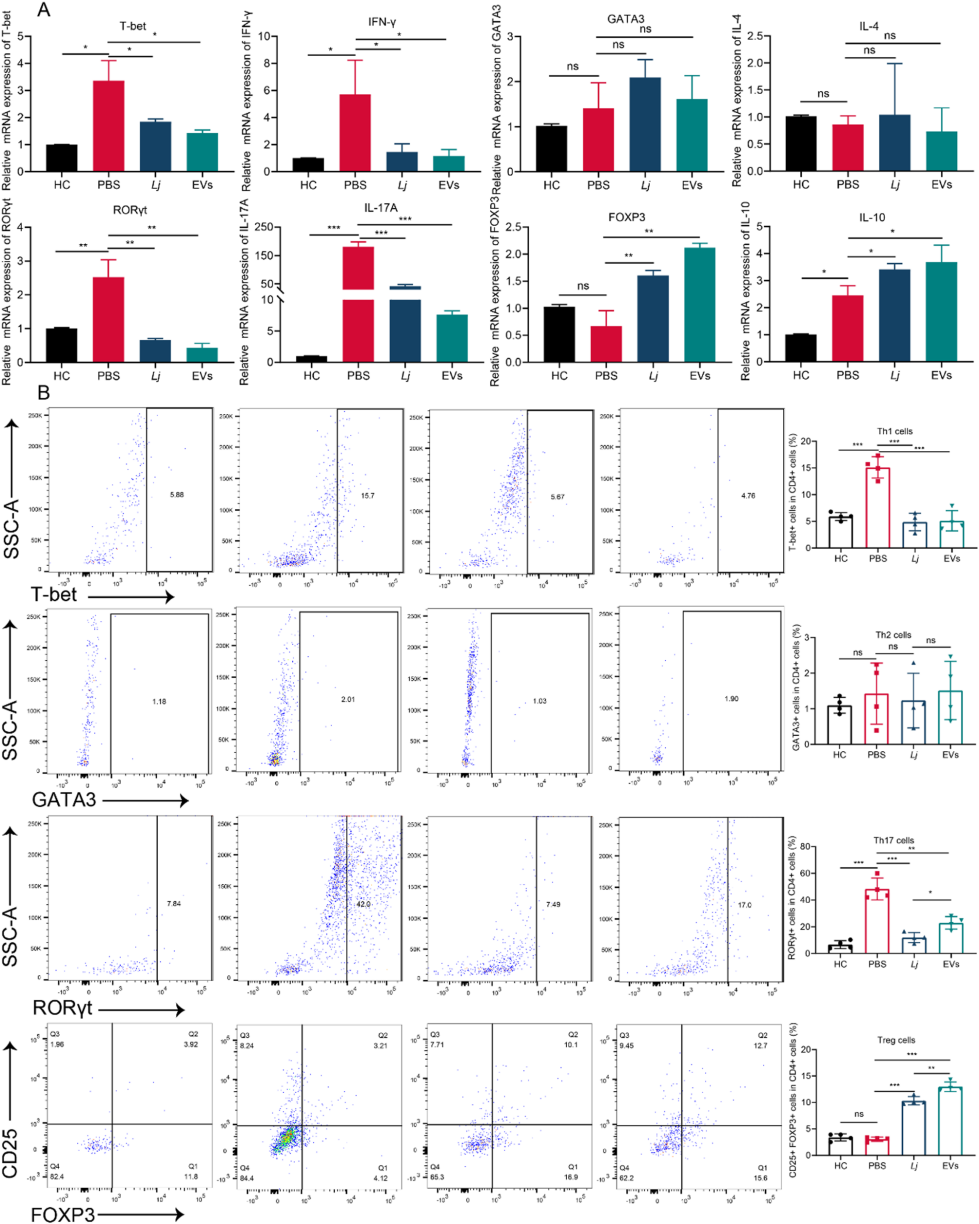
Both *L. johnsonii* and its derived EVs restored the Th17/Treg balance. (**A**) Relative mRNA expression of lineage-defining transcription factors and signature cytokines for Th1 (T-bet, IFN-γ), Th2 (GATA3, IL-4), Th17 (RORγt, IL-17 A), and Treg (FOXP3, IL-10) cells in colonic tissues from DSS-induced colitis mice following treatment with PBS, *L. johnsonii*, or EVs (*n* = 3). (**B**) Flow cytometric analysis of colonic lamina propria CD4⁺ T cell subsets, including Th1 (CD4⁺T-bet⁺), Th2 (CD4⁺GATA3⁺), Th17 (CD4⁺RORγt⁺), and Treg (CD4⁺CD25⁺FOXP3⁺) populations (n = 4). Data are presented as mean ± SD. *p < 0.05, **p < 0.01, ***p < 0.001

To comprehensively assess mucosal T cell responses in DSS-induced colitis, we profiled major CD4⁺ T cell subsets—Th1, Th2, Th17, and Treg—by evaluating lineage-defining transcription factors and signature cytokines at both the mRNA (Fig. 7A) and cellular (Fig. 7B) levels. This approach aimed to determine whether *L. johnsonii* or its EVs could modulate pro-inflammatory versus regulatory T cell polarization, thereby influencing downstream mucosal immunoglobulin transport (PIGR/FcRn) and the extent of bacterial coating by IgA, IgG, and IgM. Within the Th1/Th2 axis, DSS administration markedly increased T-bet and IFN-γ expression and elevated Th1 cell proportions, while exerting minimal effects on GATA3, IL-4, or Th2 frequency. Both *L. johnsonii* and EV treatment attenuated Th1-associated markers toward baseline, with no significant impact on Th2 cells. Pronounced changes were observed in the Th17/Treg axis. DSS challenge strongly upregulated RORγt and IL-17 A and expanded the Th17 population, concomitant with reduced FOXP3 and diminished Treg frequency, while IL-10 expression showed a slight increase. Intervention with either *L. johnsonii* or EVs significantly suppressed Th17 responses while enhancing Treg cell abundance and transcriptional markers, effectively restoring the Th17/Treg balance. Given the central role of Th17/Treg polarization in regulating mucosal antibody responses, these immune shifts prompted further investigation into antibody transport and bacterial immunoglobulin coating. We therefore next examined colonic PIGR/FcRn expression and the proportions of bacteria coated with IgA, IgG, and IgM.

### Both L. johnsonii and its derived EVs reduced the level of highly Ig-coated gut bacteria

**Fig. 8:**
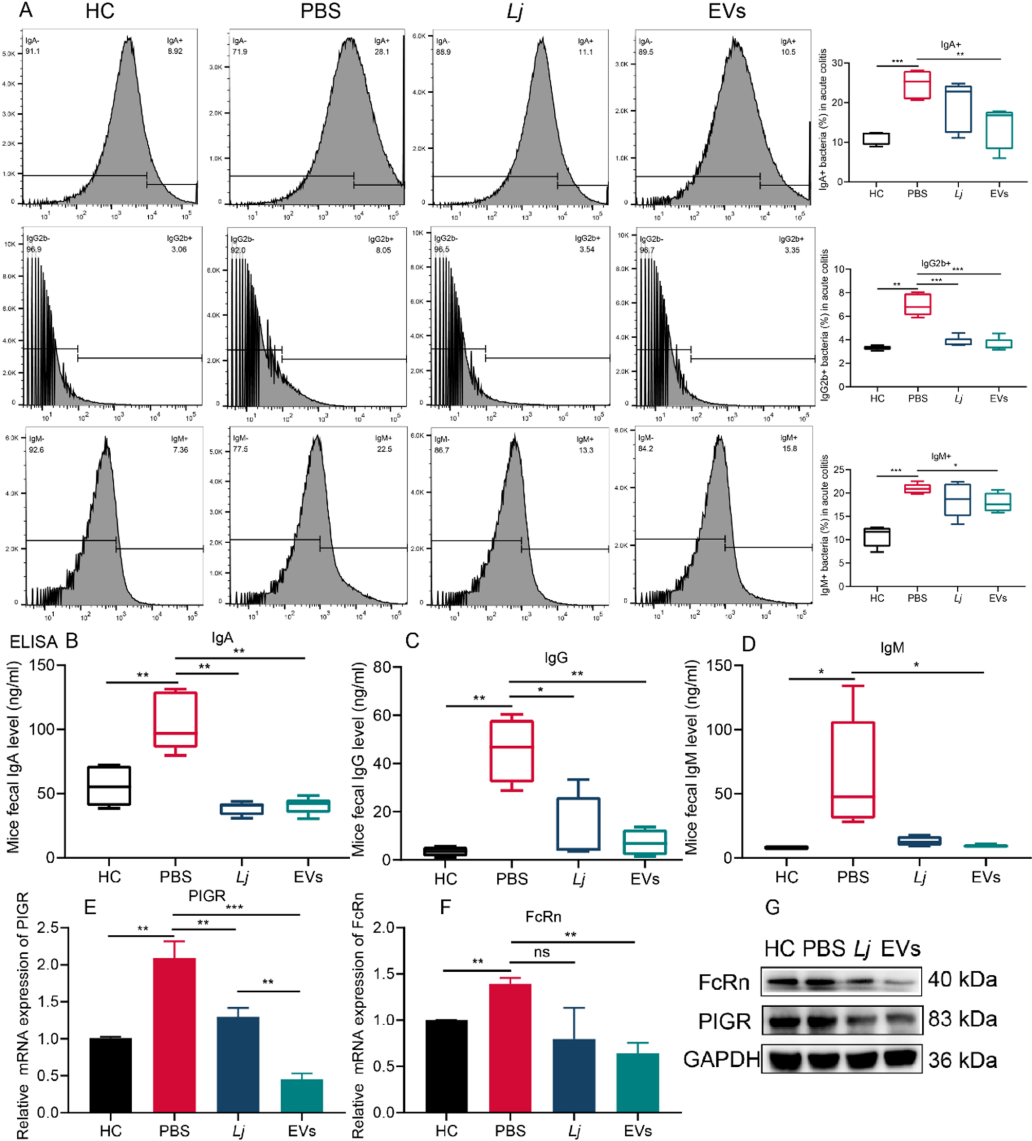
Both *L. johnsonii* and its derived EVs reduced the level of highly Ig-coated gut bacteria. (**A**) Bacterial flow cytometry analysis of highly Ig-bound bacteria in colitis mice (*n* = 5). (**B-D**) ELISA results of fecal IgA, IgG, and IgM levels in mice (*n* = 4–5). (**E-F**) mRNA expression of PIGR and FcRn in mice colon (*n* = 3). (**G**) Western blot analysis of PIGR and FcRn in mice colon. Data are expressed as mean ± SD. *p < 0.05, **p < 0.01, ***p < 0.001. Abbreviation: pIgR, polymeric immunoglobulin receptor; FcRn, neonatal Fragment crystallizable receptor

We next investigated whether the observed Th17/Treg rebalancing translated into altered mucosal antibody responses. Flow cytometry revealed that DSS-induced colitis significantly elevated the proportions of IgA⁺, IgG2b⁺, and IgM⁺ bacteria compared to healthy controls, indicating heightened immune targeting of gut microbes during inflammation. Oral administration of EVs markedly reduced the abundance of all three highly Ig-coated bacterial populations, whereas *L. johnsonii* primarily decreased IgG2b⁺ bacteria (Fig. 8A). Consistently, ELISA measurements showed elevated fecal IgA, IgG, and IgM in DSS-treated mice, which were significantly reduced by both *L. johnsonii* and EVs (Fig. 8B–D), without changes in serum immunoglobulins (Supplementary Fig. S2), indicating a localized mucosal effect. To probe the underlying mechanism, we quantified colonic expression of the immunoglobulin transporters PIGR and FcRn. qPCR and western blot analyses revealed that both *L. johnsonii* and EVs suppressed PIGR and FcRn at mRNA and protein levels, with EVs exerting a more pronounced effect (Fig. 8F–G). These findings suggest that *L. johnsonii* and its EVs attenuate epithelial Ig transcytosis, thereby reducing luminal IgA/IgG accumulation and limiting bacterial immunoglobulin coating.

### Both L. johnsonii and its derived EVs remodeled gut microbiota and amino acid metabolism

**Fig. 9:**
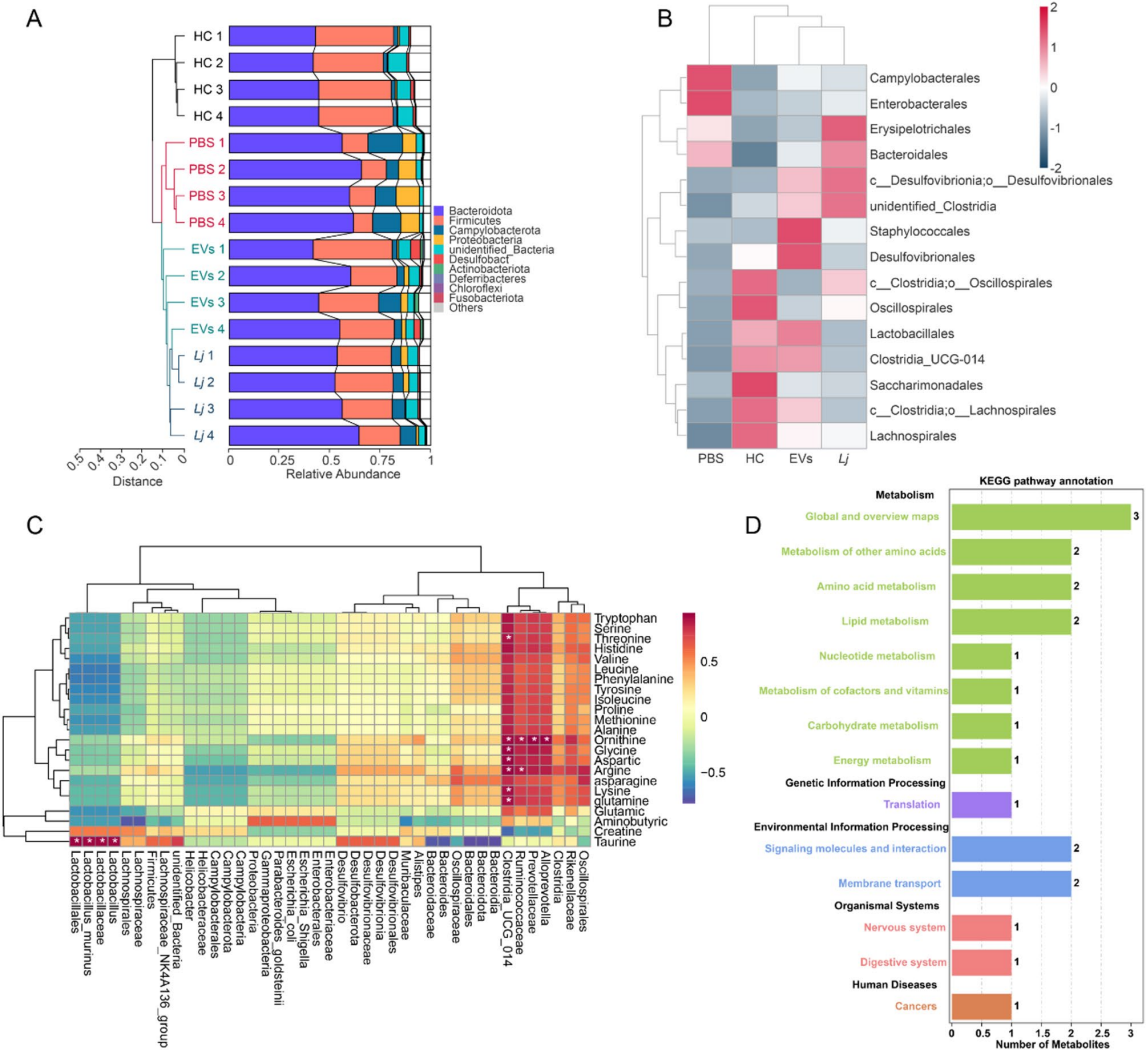
Both *L. johnsonii* and its derived EVs remodeled gut microbiota and amino acid metabolism. (**A**) UPGMA analysis of microbiota composition at the phylum level (*n* = 4). (**B**) Heatmap of relative abundance of the top 15 taxa at the order level (*n* = 5). (**C**) Pearson correlation heatmap between 16 S rRNA abundance and 23 amino acids levels. (**D**) KO enrichment analysis of taurine- and glycine-associated KEGG pathways. Abbreviation: UPGMA, unweighted pair group method with arithmetic mean.

To evaluate the effects of *L. johnsonii* and its EVs on intestinal microbial communities, 16 S rRNA gene sequencing was performed on fecal samples from DSS-induced colitis mice. At the phylum level, colitis was characterized by increased Bacteroidota, Proteobacteria, and Campylobacterota, along with a marked reduction in Firmicutes. Both interventions significantly reversed these alterations, with EVs producing a more pronounced restoration of Firmicutes and suppression of colitis-associated phyla (Fig. 9A). At the order level, beneficial taxa including Lactobacillales, Clostridia_UCG-014, and Lachnospirales were depleted in colitis mice but restored following treatment, with EVs inducing a broader and more robust recovery (Fig. 9B). While both *L. johnsonii* and EVs corrected dysbiosis, EVs uniquely promoted taxa strongly associated with taurine metabolism. Correlation analysis revealed that Lactobacillales, Lactobacillaceae, and *Lactobacillus murinus* exhibited strong positive correlations with taurine levels (*r* > 0.91, *p* < 0.05), implicating lactobacilli in taurine biosynthesis or retention (Fig. 9C). Additionally, Clostridia_UCG-014 abundance correlated with multiple amino acids, including glycine, threonine, arginine, and glutamine, which are linked to epithelial protection and immune regulation (Supplementary_out.pearson). KEGG Orthology enrichment further demonstrated that taurine was enriched in pathways related to sulfur metabolism, ABC transporters, and taurine/hypotaurine metabolism (Fig. 9D, Supplementary_need.path), and its abundance was inversely correlated with colonic PIGR and FcRn expression, key receptors mediating IgA and IgG transcytosis. Collectively, these findings indicate that *L. johnsonii* and its EVs restore gut microbial composition, while EVs specifically promote immunoregulatory amino acids—especially taurine—and suppress epithelial Ig transport pathways, thereby linking microbiota remodeling to the attenuation of dysregulated mucosal immune responses in colitis.

### Taurine supplementation reproduced the immunomodulatory and barrier-protective effects of L. johnsonii-derived EVs

**Fig. 10:**
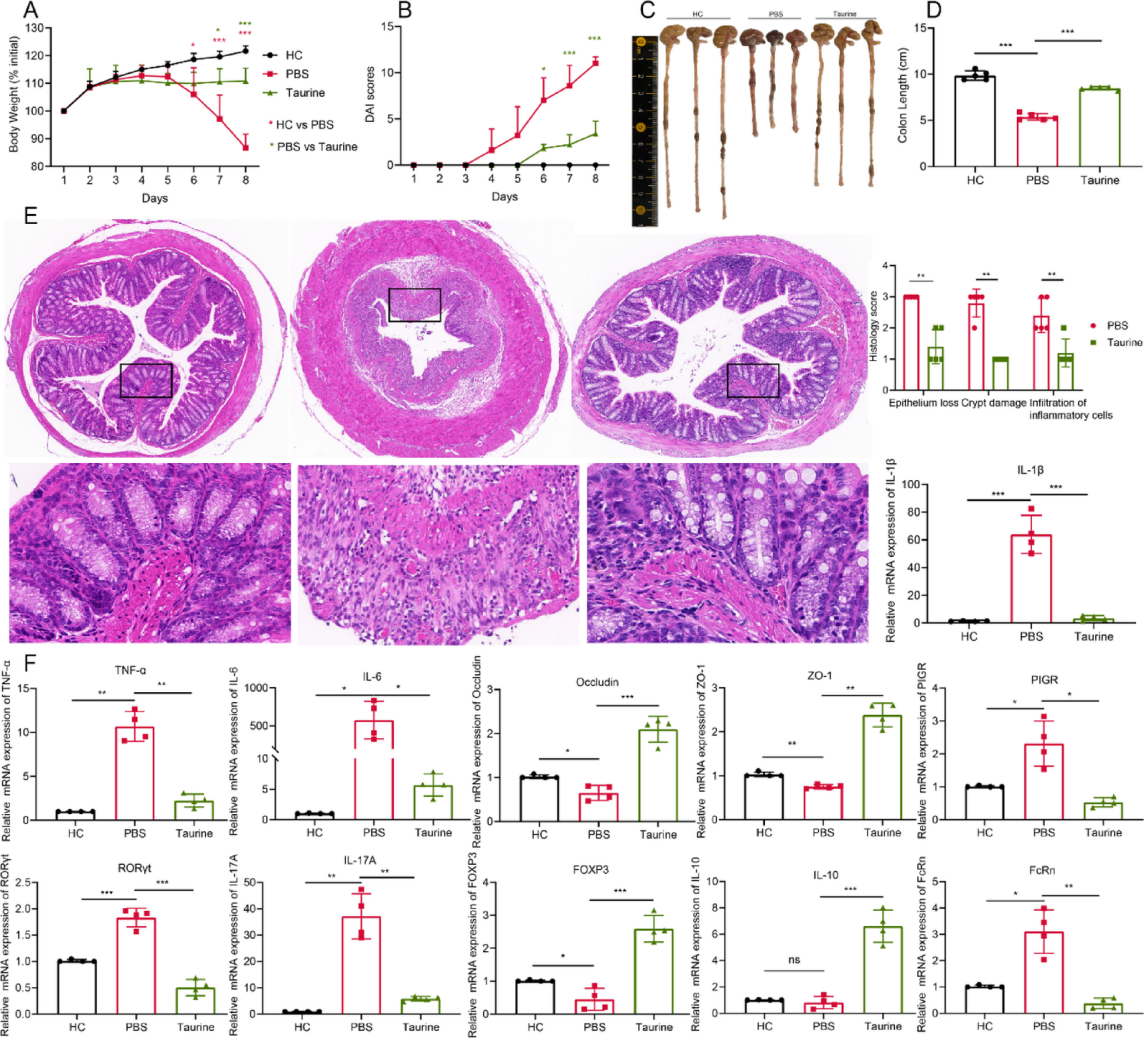
Taurine supplementation reproduced the immunomodulatory and barrier-protective effects of *L. johnsonii*-derived EVs. (**A**) Body weight changes (*n* = 5). (**B**) DAI scores (*n* = 5). (**C**) Representative images of colons. (**D**) Colon length (*n* = 5). (**E**) Representative H&E-stained colon sections (×200) with corresponding histopathological scores (*n* = 5). (**F**) Colonic mRNA expression of inflammatory cytokines, tight junction proteins, Th17/Treg-associated markers, and immunoglobulin transporters (*n* = 4). Data are presented as mean ± SD. *p < 0.05, **p < 0.01, ***p < 0.001

Given that multi-omics analysis identified taurine as the key immunoregulatory metabolite most strongly associated with EV-mediated microbiota remodeling (Fig. 9), we next investigated whether direct taurine supplementation could mimic the protective effects of EVs in DSS-induced colitis. Taurine-treated mice exhibited significantly attenuated body weight loss (Fig. 10A) and reduced DAI scores (Fig. 10B) compared to PBS-treated colitis controls. Gross examination revealed that taurine supplementation preserved colon length (Fig. 10C–D), and histological analysis demonstrated improved mucosal architecture with reduced epithelial loss, crypt damage, and inflammatory cell infiltration (Fig. 10E). At the molecular level, taurine supplementation markedly suppressed colonic expression of pro-inflammatory cytokines TNF-α, IL-6, and IL-1β, and downregulated the Th17-associated transcription factor RORγt and effector cytokine IL-17 A (Fig. 10F). Conversely, taurine increased FOXP3 expression and modestly elevated IL-10, indicating partial restoration of the Th17/Treg balance. In parallel, taurine treatment enhanced epithelial barrier integrity, as evidenced by elevated mRNA levels of tight junction proteins Occludin and ZO-1, while significantly reducing the expression of PIGR and FcRn—key receptors mediating IgA and IgG transcytosis. Collectively, these results demonstrate that taurine supplementation recapitulates the major immunomodulatory and barrier-protective effects of *L. johnsonii*-derived EVs, including suppression of inflammatory cytokines, rebalancing of the Th17/Treg axis, enhancement of barrier function, and inhibition of epithelial Ig transport pathways. These findings provide functional validation for taurine as a mechanistic link between EV-driven microbiota remodeling and attenuation of dysregulated mucosal immune responses.

## Discussion

In this study, we show that *L. johnsonii*-derived EVs restore mucosal immune homeostasis in colitis by integrating microbiota remodeling, taurine-driven metabolic regulation, and Th17/Treg rebalancing. Both *L. johnsonii* and its EVs suppressed RORγt/IL-17 A⁺ Th17 cells, expanded FOXP3⁺ Tregs, and shifted the intestinal immune milieu toward a regulatory state. EVs uniquely enriched taurine-associated taxa (Lactobacillales, Lactobacillaceae, *L. murinus*), elevating taurine levels and linking microbial changes to immunoregulatory metabolism. These upstream effects converged to downregulate colonic pIgR and FcRn, reduce luminal IgA/IgG accumulation, and limit excessive antibody coating of gut bacteria.

Our data confirm inflammation-associated colonic enrichment and epithelial association of bacterial EVs. *Lactobacillus acidophilus*-EVs coated on UiO-66-NH₂ nanoparticles have been shown to accumulate specifically in inflamed colonic tissue and undergo efficient internalization by intestinal epithelial cells in murine colitis models [28]. In agreement, we demonstrate that *L. johnsonii*-EVs are enriched in inflamed colon and closely associate with epithelial cells, underscoring their potential as an oral delivery platform for IBD therapy.

Bacterial EVs offer distinct safety and stability advantages over mammalian EVs. Unlike mesenchymal stem cell-derived EVs, which carry theoretical risks of oncogenic cargo and require stringent manufacturing controls [29, 30], bacterial EVs withstand gastric acidity and enzymatic degradation, and can be manufactured at scale without live cultures [31]. Consistently, oral administration of *L. johnsonii*-derived EVs caused no histopathological or biochemical toxicity in vivo, confirming their biocompatibility and translational potential. It is worth noting that while the relative abundances of certain orders such as Staphylococcales and Desulfovibrionales remained slightly elevated after EV treatment, these taxa comprise both pathogenic and non-pathogenic members, and such changes may represent transient ecological adjustments during recovery rather than persistent dysbiosis. Importantly, these localized shifts did not compromise the overall functional restoration of the microbiota or the improvement in host inflammatory and barrier parameters.

Functionally, both *L. johnsonii* and its EVs rebalanced the Th17/Treg axis—a pivotal regulator of mucosal antibody specificity and affinity—thereby creating an immune milieu less permissive to excessive IgA/IgG responses. Building on prior observations that IBD patients harbor numerous taxa with elevated IgA coating [32] and that UC patient-derived fecal EVs contribute to mucosal immune dysregulation [33], we show that *L. johnsonii*-EVs downregulate epithelial pIgR and FcRn, reducing luminal IgA and IgG transcytosis and normalizing bacterial immune targeting. Notably, while both interventions lowered the prevalence of highly Ig-coated bacteria, EVs achieved broader suppression across IgA, IgG, and IgM, underscoring their superior capacity to constrain aberrant antibody–microbiota interactions.

Microbial EV therapy orchestrates concurrent remodeling of gut microbiota composition and host amino acid metabolism. Prior studies have shown that dietary taurine supplementation can restore *Lactobacillus* abundance and ameliorate colitis in rodents [34, 35], together with evidence that *Bifidobacterium longum*-derived EVs modulate short-chain fatty acid profiles to dampen inflammation [36]. Extending these findings, our multi-omics analysis identifies EV-driven enrichment of taurine-associated taxa—most notably Lactobacillales, Lactobacillaceae, and *Lactobacillus murinus*—alongside upregulation of taurine biosynthesis and metabolic pathways. This microbial–metabolic shift is mechanistically linked to suppression of epithelial Ig transport (pIgR/FcRn) and mitigation of dysregulated mucosal immunity. Importantly, direct taurine supplementation phenocopied the immunomodulatory and barrier-protective effects of EVs, including Th17/Treg rebalancing, downregulation of pIgR/FcRn, and enhancement of epithelial integrity, thereby validating taurine as a key effector within the EV–microbiota–immune regulatory axis.

Our findings position *L. johnsonii*-derived EVs as a multifunctional, gut-directed therapeutic platform that integrates immune modulation, microbiota remodeling, and metabolic regulation. By reprogramming the Th17/Treg axis, enriching taurine-associated bacterial taxa, and suppressing epithelial Ig transport, these vesicles attenuate dysregulated antibody–microbiota interactions and restore mucosal immune balance. Their scalability, gastrointestinal stability, and inflammation-associated colonic enrichment support their translational feasibility and suitability for oral delivery, offering a patient-friendly approach for long-term IBD management. The mechanistic link between EV-driven microbiota–metabolite changes and T cell polarization further highlights opportunities to combine EV therapy with dietary or metabolic interventions for synergistic benefit. Future work should optimize dosing strategies, define vesicular cargo mediating Ig transporter regulation, and evaluate durability and safety in advanced preclinical models, paving the way for clinical translation of these microbial nanotherapeutics.

## Conclusions

In summary, this study identifies heightened immunoglobulin coating of gut microbiota as a prominent feature of active IBD, closely associated with disease severity and inflammatory burden. *L. johnsonii*, a beneficial commensal markedly depleted in UC, and its EVs both attenuated experimental colitis; however, EVs exhibited superior stability, colonic targeting, and therapeutic potency. Mechanistically, *L. johnsonii*-derived EVs orchestrated a coordinated EV–taurine–Th17/Treg–pIgR/FcRn–IgA/IgG axis, characterized by rebalancing of mucosal T cell responses, enrichment of taurine-associated bacterial taxa, suppression of epithelial immunoglobulin transport, and reduction of dysregulated antibody–microbiota interactions. By integrating microbial, metabolic, and immune modulation, these microbial nanovesicles represent a promising, orally deliverable platform for next-generation gut-focused therapeutics in inflammatory bowel disease.

## Supplementary Information

Supplementary Material 1.

Supplementary Material 2.

Supplementary Material 3.

## Data Availability

No datasets were generated or analysed during the current study.

## Abbreviations

IBD: Inflammatory bowel disease
UC: Ulcerative colitis
CD: Crohn’s disease
Ig: Immunoglobulin
Treg: regulatory T cells
pIgR: polymeric immunoglobulin receptor
FcRn: neonatal Fragment crystallizable receptor
DSS: Dextran sulfate sodium
EVs: Extracellular vesicles
IHC: Immunohistochemical
TEM: Transmission electron microscopy
MPO: Myeloperoxidase
OTUs: Operational taxonomic units
LC-MS: Liquid chromatography‒mass spectrometry
CFUs: Colony forming units
DAI: Disease activity index
CK-MB: Creatine kinase-MB
ALT: Alanine aminotransferase
AST: Aspartate aminotransferase
ESR: Erythrocyte sedimentation rate
UPGMA: Unweighted pair group method with arithmetic mean
LEfSe: Linear discriminant analysis effect size

## References

[1] Caruso R, Lo BC, Nunez G. Host-microbiota interactions in inflammatory bowel disease. Nat Rev Immunol. 2020;20:411–26. 32005980 10.1038/s41577-019-0268-7

[2] de Souza HS, Fiocchi C. Immunopathogenesis of IBD: current state of the art. Nat Rev Gastroenterol Hepatol. 2016;13:13–27. 26627550 10.1038/nrgastro.2015.186

[3] Janzon A, Goodrich JK, Koren O, Waters JL, Ley RE. Interactions between the gut microbiome and mucosal immunoglobulins A, M, and G in the developing infant gut. mSystems. 2019. 10.1128/mSystems.00612-19. 31771976 10.1128/mSystems.00612-19 PMC6880043

[4] Augustine T, Murugesan S, Badri F, Gentilcore G, Grivel JC, Akobeng A, Elawad M, Adeli M, Al Khodor S, van Panhuys N. Immunoglobulin-coating patterns reveal altered humoral responses to gut bacteria in pediatric cow milk allergies. J Transl Med. 2024;22:1021. 39533360 10.1186/s12967-024-05850-z PMC11558889

[5] Bunker JJ, Bendelac A. IgA responses to microbiota. Immunity. 2018;49:211–24. 30134201 10.1016/j.immuni.2018.08.011 PMC6107312

[6] Li H, Limenitakis JP, Greiff V, Yilmaz B, Scharen O, Urbaniak C, et al. Mucosal or systemic microbiota exposures shape the B cell repertoire. Nature. 2020;584:274–8. 32760003 10.1038/s41586-020-2564-6

[7] Vergani S, Muleta KG, Da Silva C, Doyle A, Kristiansen TA, Sodini S, Krausse N, Montano G, Kotarsky K, Nakawesi J, et al. A self-sustaining layer of early-life-origin B cells drives steady-state IgA responses in the adult gut. Immunity. 2022;55:1829–e18421826. 36115337 10.1016/j.immuni.2022.08.018

[8] Hirota K, Turner JE, Villa M, Duarte JH, Demengeot J, Steinmetz OM, et al. Plasticity of Th17 cells in Peyer’s patches is responsible for the induction of T cell-dependent IgA responses. Nat Immunol. 2013;14:372–9. 23475182 10.1038/ni.2552 PMC3672955

[9] Zeng MY, Cisalpino D, Varadarajan S, Hellman J, Warren HS, Cascalho M, et al. Gut microbiota-induced immunoglobulin G controls systemic infection by symbiotic bacteria and pathogens. Immunity. 2016;44:647–58. 26944199 10.1016/j.immuni.2016.02.006 PMC4794373

[10] Moor K, Diard M, Sellin ME, Felmy B, Wotzka SY, Toska A, et al. High-avidity IgA protects the intestine by enchaining growing bacteria. Nature. 2017;544:498–502. 28405025 10.1038/nature22058

[11] Bunker JJ, Erickson SA, Flynn TM, Henry C, Koval JC, Meisel M, et al. Natural polyreactive IgA antibodies coat the intestinal microbiota. Science. 2017;358(6361) . 10.1126/science.aan6619 28971969 10.1126/science.aan6619 PMC5790183

[12] Chen K, Magri G, Grasset EK, Cerutti A. Rethinking mucosal antibody responses: igm, IgG and IgD join IgA. Nat Rev Immunol. 2020;20:427–41. 32015473 10.1038/s41577-019-0261-1 PMC10262260

[13] Armstrong H, Alipour M, Valcheva R, Bording-Jorgensen M, Jovel J, Zaidi D, et al. Host immunoglobulin G selectively identifies pathobionts in pediatric inflammatory bowel diseases. Microbiome. 2019;7:1. 30606251 10.1186/s40168-018-0604-3 PMC6317230

[14] Rengarajan S, Vivio EE, Parkes M, Peterson DA, Roberson EDO, Newberry RD, et al. Dynamic immunoglobulin responses to gut bacteria during inflammatory bowel disease. Gut Microbes. 2020;11:405–20. 31203722 10.1080/19490976.2019.1626683 PMC7524373

[15] Kamada N, Sakamoto K, Seo SU, Zeng MY, Kim YG, Cascalho M, et al. Humoral immunity in the gut selectively targets phenotypically virulent attaching-and-effacing bacteria for intraluminal elimination. Cell Host Microbe. 2015;17:617–27. 25936799 10.1016/j.chom.2015.04.001 PMC4433422

[16] Palm NW, de Zoete MR, Cullen TW, Barry NA, Stefanowski J, Hao L, Degnan PH, Hu J, Peter I, Zhang W, et al. Immunoglobulin A coating identifies colitogenic bacteria in inflammatory bowel disease. Cell. 2014;158:1000–10. 25171403 10.1016/j.cell.2014.08.006 PMC4174347

[17] Díaz-Garrido N, Badia J, Baldomà L. Microbiota-derived extracellular vesicles in interkingdom communication in the gut. J Extracell Vesicles. 2021;10:e12161. 34738337 10.1002/jev2.12161 PMC8568775

[18] Gill S, Catchpole R, Forterre P. Extracellular membrane vesicles in the three domains of life and beyond. FEMS Microbiol Rev. 2019;43:273–303. 30476045 10.1093/femsre/fuy042 PMC6524685

[19] Toyofuku M, Schild S, Kaparakis-Liaskos M, Eberl L. Composition and functions of bacterial membrane vesicles. Nat Rev Microbiol. 2023;21:415–30. 36932221 10.1038/s41579-023-00875-5

[20] Yue N, Zhao H, Hu P, Zhang Y, Tian C, Kong C, et al. Real-world of *Limosilactobacillus reuteri* in mitigation of acute experimental colitis. J Nanobiotechnol. 2025;23:65. 10.1186/s12951-025-03158-8 PMC11783912 39891249

[21] Kim W, Lee EJ, Bae IH, Myoung K, Kim ST, Park PJ, et al. *Lactobacillus plantarum*-derived extracellular vesicles induce anti-inflammatory M2 macrophage polarization in vitro. J Extracell Vesicles. 2020;9:1793514. 32944181 10.1080/20013078.2020.1793514 PMC7480564

[22] Tao S, Fan J, Li J, Wu Z, Yao Y, Wang Z, et al. Extracellular vesicles derived from *Lactobacillus johnsonii* promote gut barrier homeostasis by enhancing M2 macrophage polarization. J Adv Res. 2025;69:545–63. 38508446 10.1016/j.jare.2024.03.011 PMC11954842

[23] Moayyedi P, Surette MG, Kim PT, Libertucci J, Wolfe M, Onischi C, Armstrong D, Marshall JK, Kassam Z, Reinisch W, Lee CH. Fecal microbiota transplantation induces remission in patients with active ulcerative colitis in a randomized controlled trial. Gastroenterology. 2015;149:102–e109106. 25857665 10.1053/j.gastro.2015.04.001

[24] Zhao H, Zhou Y, Xu J, Zhang Y, Wang H, Zhao C, Huang H, Yang J, Huang C, Li Y, et al. Short-chain fatty acid-producing bacterial strains attenuate experimental ulcerative colitis by promoting M2 macrophage polarization via JAK/STAT3/FOXO3 axis inactivation. J Transl Med. 2024;22:369. 38637862 10.1186/s12967-024-05122-w PMC11025230

[25] Zhao H, Peng Y, Cai X, Zhou Y, Zhou Y, Huang H, et al. Genome insights of *Enterococcus raffinosus* CX012922, isolated from the feces of a Crohn’s disease patient. Gut Pathog. 2021;13:71. 34876224 10.1186/s13099-021-00468-8 PMC8650288

[26] Zhao H, Wang J, Peng Y, Cai X, Liu Y, Huang W, et al. Genomic insights from *paraclostridium bifermentans* HD0315_2: general features and pathogenic potential. Front Microbiol. 2022;13:928153. 36090102 10.3389/fmicb.2022.928153 PMC9449513

[27] Wang X, Lin S, Wang L, Cao Z, Zhang M, Zhang Y, et al. Versatility of bacterial outer membrane vesicles in regulating intestinal homeostasis. Sci Adv. 2023;9:eade5079. 36921043 10.1126/sciadv.ade5079 PMC10017049

[28] Cui C, Tang J, Chen J, Zhang B, Li R, Zhang Q, et al. *Lactobacillus acidophilus* extracellular vesicles-coated UiO-66-NH(2)@siRNA nanoparticles for ulcerative colitis targeted gene therapy and gut microbiota modulation. J Nanobiotechnol. 2025;23(1):301. 10.1186/s12951-025-03376-0 PMC12007195 40247297

[29] Ocansey DKW, Qiu W, Wang J, Yan Y, Qian H, Zhang X, Xu W, Mao F. The Achievements and Challenges of Mesenchymal Stem Cell-Based Therapy in Inflammatory Bowel Disease and Its Associated Colorectal Cancer. *Stem Cells Int* 2020, 2020:7819824. 10.1155/2020/7819824 PMC7104387 32256612

[30] Giovannelli L, Bari E, Jommi C, Tartara F, Armocida D, Garbossa D, et al. Mesenchymal stem cell secretome and extracellular vesicles for neurodegenerative diseases: risk-benefit profile and next steps for the market access. Bioact Mater. 2023;29:16–35. 37456581 10.1016/j.bioactmat.2023.06.013 PMC10338239

[31] Wang S, Luo J, Wang H, Chen T, Sun J, Xi Q, et al. Extracellular vesicles: a crucial player in the intestinal microenvironment and beyond. Int J Mol Sci. 2024;25(6):3478 . 10.3390/ijms25063478 38542448 10.3390/ijms25063478 PMC10970531

[32] van Gogh M, Louwers JM, Celli A, Gräve S, Viveen MC, Bosch S, de Boer NKH, Verheijden RJ, Suijkerbuijk KPM, Brand EC, et al. Next-generation IgA-SEQ allows for high-throughput, anaerobic, and metagenomic assessment of IgA-coated bacteria. Microbiome. 2024;12:211. 39434178 10.1186/s40168-024-01923-9 PMC11492651

[33] Thapa HB, Passegger CA, Fleischhacker D, Kohl P, Chen YC, Kalawong R, Tam-Amersdorfer C, Gerstorfer MR, Strahlhofer J, Schild-Prüfert K, et al. Enrichment of human IgA-coated bacterial vesicles in ulcerative colitis as a driver of inflammation. Nat Commun. 2025;16:3995. 40301356 10.1038/s41467-025-59354-5 PMC12041585

[34] Qian W, Li M, Yu L, Tian F, Zhao J, Zhai Q. Effects of taurine on gut microbiota homeostasis: an evaluation based on two models of gut dysbiosis. Biomedicines. 2023;11(4):1048 . 10.3390/biomedicines11041048 37189666 10.3390/biomedicines11041048 PMC10135931

[35] Zheng J, Zhang J, Zhou Y, Zhang D, Guo H, Li B, et al. Taurine alleviates experimental colitis by enhancing intestinal barrier function and inhibiting inflammatory response through TLR4/NF-κB signaling. J Agric Food Chem. 2024;72:12119–29. 38761152 10.1021/acs.jafc.4c00662

[36] Nie X, Li Q, Ji H, Zhang S, Wang Y, Xie J, et al. *Bifidobacterium longum* NSP001-derived extracellular vesicles ameliorate ulcerative colitis by modulating T cell responses in gut microbiota-(in)dependent manners. NPJ Biofilms Microbiomes. 2025;11:27. 39929833 10.1038/s41522-025-00663-4 PMC11811157

