# Supplementary material for: Lactobacillus johnsonii-derived extracellular vesicles restore mucosal immunity via taurine-linked Th17/Treg and IgA/IgG regulation in colitis
Source: J Nanobiotechnology. 2025 Sep 29;23:612. doi: 10.1186/s12951-025-03702-6 (PMC12482724; doi:10.1186/s12951-025-03702-6)

***Lactobacillus johnsonii*-Derived Extracellular Vesicles Restore Mucosal Immunity via Taurine-Linked Th17/Treg and IgA/IgG Regulation in Colitis**

**Authors:**

Hailan Zhao^1,2†^, Ningning Yue^1†^, Zhiliang Mai^1^, Yuan Zhang^4^, Chengmei Tian^5^, Chen Kong^1^, Longbin Huang^1^, Ruiyue Shi^1^, Yujie Liang^6^, Jun Yao^1^, Yuqiang Nie^3*^, Defeng Li^1*^, Biao Nie^2*^, Lisheng Wang^1*^

**Affiliations:**

1. Department of Gastroenterology, Shenzhen People’s Hospital (The First Affiliated Hospital, Southern University of Science and Technology; The Second Clinical Medical College, Jinan University), Shenzhen 518020, Guangdong, China
2. The First Affiliated Hospital, Jinan University, Guangzhou 510630, Guangdong, China
3. Department of Gastroenterology and Hepatology, Guangzhou First People’s Hospital, the Second Affiliated Hospital, School of Medicine, South China University of Technology, Guangzhou 510006, Guangdong, China.
4. Department of Medical Administration, Huizhou Institute of Occupational Diseases Control and Prevention, Huizhou 516008, Guangdong, China
5. Department of Emergency, Shenzhen People’s Hospital (The First Affiliated Hospital, Southern University of Science and Technology; The Second Clinical Medical College, Jinan University), Shenzhen 518020, Guangdong, China
6. Department of Child and Adolescent Psychiatry, Shenzhen Institute of Mental Health, Shenzhen Kangning Hospital, Shenzhen 518020, Guangdong, China

Supplementary Table 1. Primer sequence

| Primer | Forward Primer(5’ to 3’) | Reverse Primer(5’ to 3’) |
| --- | --- | --- |
| m (IL-1β) | AGAGCATCCAGCTTCAAATCTC | CAGTTGTCTAATGGGAACGTCA |
| m (TNF-α) | TTAGAAAGGGGATTATGGCTCA | ACTCTCCCTTTGCAGAACTCAG |
| m (IL-6) | GTTGCCTTCTTGGGACTGATG | ATTGCCATTGCACAACTCTTT |
| m (IL-12) | ACGAGAGTTGCCTGGCTACTAG | CCTCATAGATGCTACCAAGGCAC |
| m (IL-23) | CATGCTAGCCTGGAACGCACAT | ACTGGCTGTTGTCCTTGAGTCC |
| m (Claudin 1) | GGACTGTGGATGTCCTGCGTTT | GCCAATTACCATCAAGGCTCGG |
| m (Muc2) | AGGGCTCGGAACTCCAGAAA | CCAGGGAATCGGTAGACATCG |
| m (Occludin) | TGGCAAGCGATCATACCCAGAG | CTGCCTGAAGTCATCCACACTC |
| m (ZO-1) | GTTGGTACGGTGCCCTGAAAGA | GCTGACAGGTAGGACAGACGAT |
| m (PIGR) | CACTGTCATCCTCAACCAGCTC | TTCTGTGGCGTCACCTCAAGGT |
| m (FcRn) | CATTGCTGGAGGTCAAACGTGG | CGATTCCAACCACAGGCACAGA |
| m (T-bet) | CCACCTGTTGTGGTCCAAGTTC | CCACAAACATCCTGTAATGGCTTG |
| m (IFN-γ) | CAGCAACAGCAAGGCGAAAAAGG | TTTCCGCTTCCTGAGGCTGGAT |
| m (GATA3) | CCTCTGGAGGAGGAACGCTAAT | GTTTCGGGTCTGGATGCCTTCT |
| m (IL-4) | ATCATCGGCATTTTGAACGAGGTC | ACCTTGGAAGCCCTACAGACGA |
| m (RORγt) | GTGGAGTTTGCCAAGCGGCTTT | CCTGCACATTCTGACTAGGACG |
| m (IL-17A) | CAGACTACCTCAACCGTTCCAC | TCCAGCTTTCCCTCCGCATTGA |
| m (FOXP3) | CCTGGTTGTGAGAAGGTCTTCG | TGCTCCAGAGACTGCACCACTT |
| m (IL-10) | GCTCTTACTGACTGGCATGAG | CGCAGCTCTAGGAGCATGTG |

Supplementary Table 2. Antibodies used for flow cytometric analysis

| Marker | Species Reactivity | Fluorochrome | Supplier / Catalog No. |
| --- | --- | --- | --- |
| CD45 | anti-mouse | FITC | BioLegend / 157608 |
| CD3 | anti-mouse | APC/Cy7 | BioLegend / 100221 |
| CD4 | anti-mouse | BB700 | BD Biosciences / 566407 |
| CD25 | anti-mouse | APC | BioLegend / 101909 |
| RORγt | anti-mouse | BV421 | BD Biosciences / 562894 |
| FOXP3 | anti-mouse | PE | BioLegend / 126403 |
| T-bet | anti-mouse | BV421 | BioLegend / 644815 |
| GATA3 | anti-mouse | PE | BioLegend / 653803 |

Supplementary Table 3. Nucleotide sequence accession number of *L. johnsonii* ATCC 33200

| Nucleotide sequence accession number |
| --- |
| TACGGGAGGCAGCAGTAGGGAATCTTCCACAATGGACGAAAGTCTGATGGAGCAACGCCGCGTGAGTGAAGAAGGGTTTCGGCTCGTAAAGCTCTGTTGGTAGTGAAGAAAGATAGAGGTAGTAACTGGCCTTTATTTGACGGTAATTACTTAGAAAGTCACGGCTAACTACGTGCCAGCAGCCGCGGTAATACGTAGGTGGCAAGCGTTGTCCGGATTTATTGGGCGTAAAGCGAGTGCAGGCGGTTCAATAAGTCTGATGTGAAAGCCTTCGGCTCAACCGGAGAATTGCATCAGAAACTGTTGAACTTGAGTGCAGAAGAGGAGAGTGGAACTCCATGTGTAGCGGTGGAATGCGTAGATATATGGAAGAACACCAGTGGCGAAGGCGGCTCTCTGGTCTGCAACTGACGCTGAGGCTCGAAAGCATGGGTAGCGAACAGGATTAGATACCCTGGTAGTCCATGCCGTAAACGATGAGTGCTAAGTGTTGGGAGGTTTCCGCCTCTCAGTGCTGCAGCTAACGCATTAAGCACTCCGCCTGGGGAGTACGACCGCAAGGTTGAAACTCAAAGGAATTGACGGGGGCCCGCACAAGCGGTGGAGCATGTGGTTTAATTCGAAGCAACGCGAAGAACCTTACCAGGTCTTGACATCCAGTGCAAACCTAAGAGATTAGGTGTTCCCTTCGGGGACGCTGAGACAGGTGGTGCATGGCTGTCGTCAGCTCGTGTCGTGAGATGTTGGGTTAAGTCCCGCAACGAGCGCAACCCTTGTCATTAGTTGCCATCATTAAGTTGGGCACTCTAATGAGACTGCCGGTGACAAACCGGAGGAAGGTGGGGATGACGTCAAGTCATCATGCCCCTTATGACCTGGGCTACACACGTGCTACAATGGACGGTACAACGAGAAGCGAACCTGCGAAGGCAAGCGGATCTCTTAAAGCCGTTCTCAGTTCGGACTGTAGGCTGCAACTCGCCTACACGAAGCTGGAATCGCTAGTAATCGCGGATCAGCACGCCGCGGTGAATACGTTCCCGGGCCTTGTACACACCGCCCGTCACACCATGAGAGTCTGTAACACCCAAAGCCGGTGGGATAACCTTTATAGGAGTCAGCCGTCTAAG |

**Supplementary Figures**

**Supplementary Fig. S1 Zeta Potential (mV) of *L. johnsonii*-EVs**


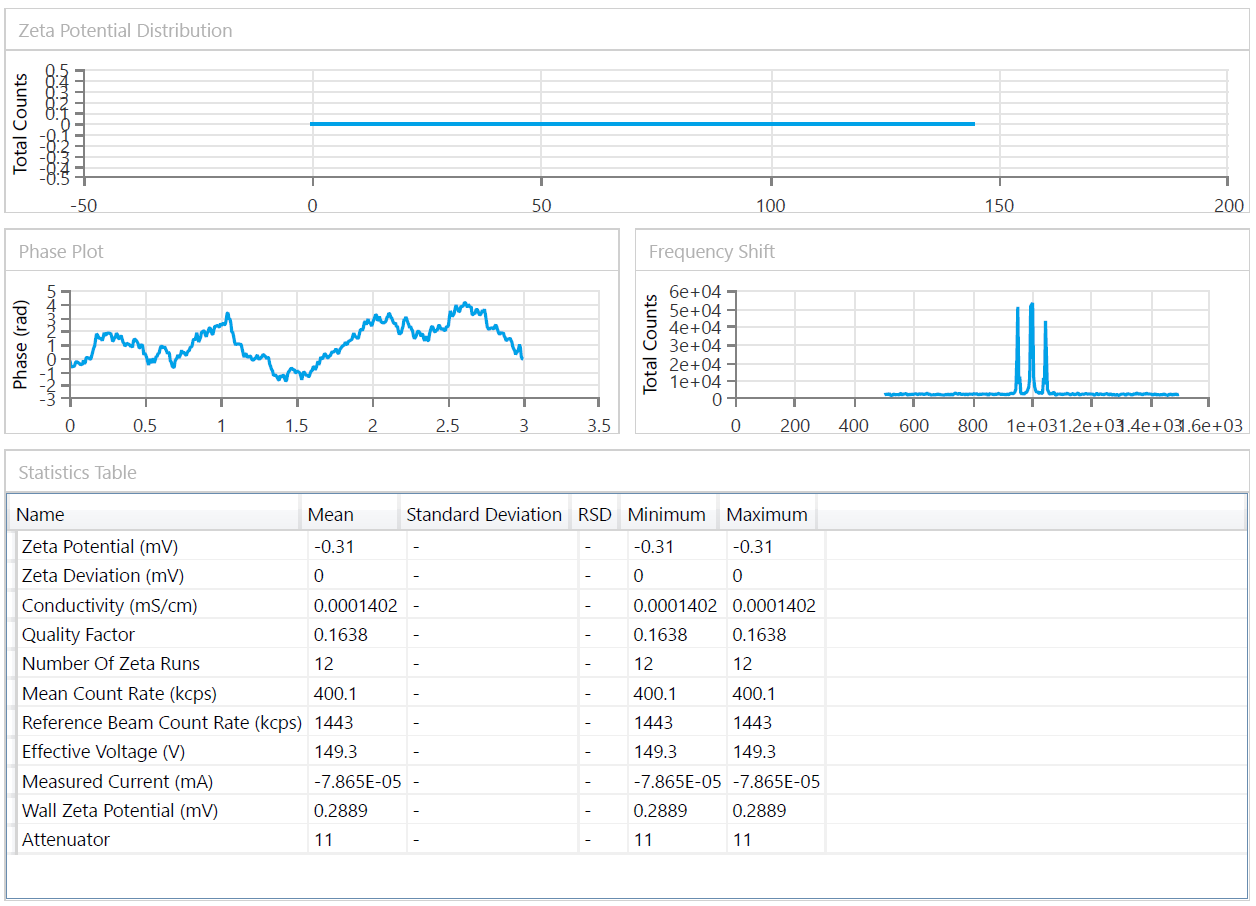


**Supplementary Fig. S2**

The ELISA results of serum IgA (**a**), IgG (**b**), IgM (**c**) levels in colitis mice (n = 4-5).


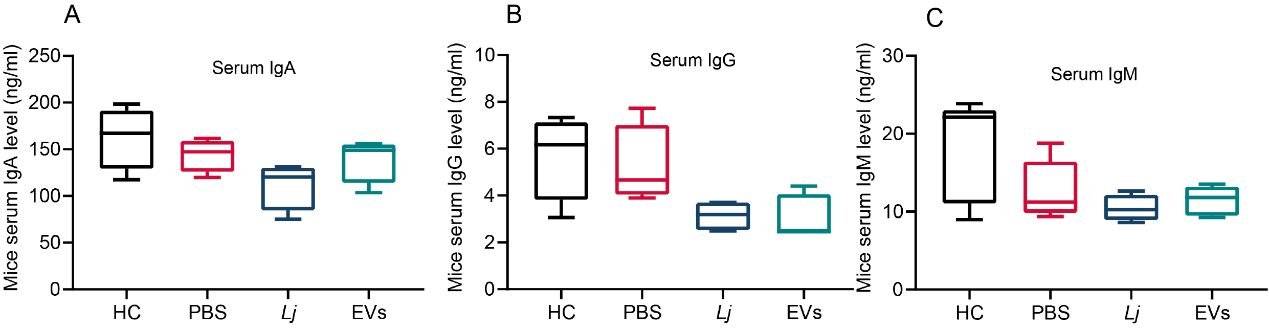

Supplement: Supplementary file 3 — Supplementary Material 3. [file 12951_2025_3702_MOESM3_ESM.docx]
